# Identification and Expression Profiling of the *HSP70* Gene Family in Melon Under Combined High Temperature and Humidity Stress

**DOI:** 10.3390/genes17101257

**Published:** 2026-10-11

**Authors:** Jinyang Weng, Zixin Wang, Muhammad Khalid Hameed, Asad Rehman, Jiahui Shao, Can Geng, Pengli Li

**Affiliations:** 1 School of Modern Agriculture, Jiangsu Agri-Animal Husbandry Vocational College, Taizhou 225300, China; wengjinyang@jsahvc.edu.cn (J.W.);; 2 School of Agriculture and Biology, Shanghai Jiao Tong University, Shanghai 200240, China; shuiwybb@sjtu.edu.cn (Z.W.); asadrehman@sjtu.edu.cn (A.R.)

**Keywords:** melon, *CmHSP70* gene family, expression analysis, high temperature and humidity stress, transcriptome

## Abstract

Background/Objectives: Heat-shock protein 70 is a key plant molecular chaperone whose family response to combined high temperature and humidity stress in melon remains uncharacterized. This study aimed to characterize the *CmHSP70* family genome-wide and compare its expression dynamics between cultivars with contrasting responses to HTH stress. Methods: The *CmHSP70* genes in melon were identified via bioinformatic analysis, with characterization covering phylogeny, gene structure, cis-elements, synteny, and selection pressure. Expression profiles under HTH stress were compared between the tolerant cultivar JD versus the sensitive cultivar JTL across 0 to 3 days using transcriptome with qRT-PCR validation, supplemented by three-dimensional (3D) structure prediction plus protein interaction network analysis. Results: The 16 CmHSP70 proteins were predicted to show diverse subcellular localizations, predominantly in the cytoplasm, endoplasmic reticulum (ER), and chloroplasts, and were grouped into five subfamilies with closest homology to cucurbit crops. Cis-acting element analysis showed that the promoters of *CmHSP70* genes contain various stress-responsive and hormone-responsive elements, implying their broad roles in stress adaptation. Gene duplication analysis revealed only one segmental duplication event and no tandem duplications in the *CmHSP70* family, indicating limited expansion during melon evolution. Ka/Ks ratios below 1 for all paralogous pairs further suggest strong purifying selection and highly conserved protein functions. Furthermore, expression profiling under high temperature and humidity (HTH) stress identified *CmHSP70-4*, *-7*, *-8*, and *-13* as key candidates, showing sustained upregulation through day 3 in the tolerant cultivar but transient induction followed by decline in the sensitive cultivar. Predicted 3D structures and a STRING-based protein–protein interaction network further highlighted these four genes within the predicted chaperone interaction framework. Conclusions: This study provides candidate gene resources for future functional validation and breeding of HTH-tolerant melon and other cucurbit crops. The association between sustained *CmHSP70* induction and the tolerant cultivar response offers a working hypothesis for molecular breeding under climate change.

## 1. Introduction

As global warming progresses, extreme heat episodes are becoming more frequent and prolonged, leaving crops vulnerable to combined HTH stress. Such stress damages the photosynthetic machinery, triggers ROS overaccumulation, and disrupts cell membranes, all of which suppress growth and lower yield [1]. Over evolutionary time, plants have built a chaperone-based defense network in which heat-shock proteins (HSPs) occupy a central position. These factors refold denatured polypeptides, curb aggregation of misfolded species, and preserve proteome stability during adverse conditions [2,3,4]. Based on their molecular weight features, heat-shock proteins falls into five principal subfamilies: sHSP, HSP100, HSP90, HSP70, and HSP60, whose members function as key molecular chaperones under stress conditions [5]. Of all HSP subgroups, HSP70 represents an evolutionarily conserved and intensively investigated protein family. Relying on the joint action of the N-terminal ATPase domain and the C-terminal substrate-binding domain, HSP70 carries out ATP-dependent chaperone activity to maintain cellular protein homeostasis [3], and it is also considered a vital regulator participating in plant adaptive responses to multiple abiotic stresses exemplified by heat and drought [6].

Plant HSP70 isoforms are not confined to a single site but are distributed across several organelles, including cytosol, chloroplasts, mitochondria, nucleus, and endoplasmic reticulum, where they individually sustain protein homeostasis in their respective cellular microenvironments [5,7]. In addition to abiotic stress responses, HSP70s participate in plant immunity [8] and seed development [9]. The growing availability of whole-genome sequences has enabled genome-wide profiling of *HSP70* across numerous crops and model plants, among them cotton [10], tobacco [11], wheat [12], maize [13], radish [14], *Brassica rapa* [15], tomato [16], and potato [17]. These studies revealed that despite high protein sequence conservation, HSP70 family size varies considerably across species, ranging from 18 in *Arabidopsis* [18] to 61 in tobacco [11], and that such variation is linked with lineage-specific whole genome and segmental replication events [10,16]. In cucurbit crops, 12 *ClHSP70* genes were identified in watermelon, showing differential expression under ABA, cold, and drought stress with all duplicated pairs under purifying selection [19], while under drought stress in pumpkin *HSP70* genes showed significant enrichment [20]. In melon (*Cucumis melo* L.), such genome-wide studies of *CmHSP70* genes have not been reported, especially regarding the temporal expression dynamics triggered by combined HTH stress.

Melon is an economically valuable cucurbit crop originating from Africa, grown worldwide and has diversified into numerous cultivar groups over the course of its long domestication history, reflecting its considerable economic importance among cucurbit crops [21]. In cultivation in southern China, melon plants frequently encounter combined HTH stress during the growing season, resulting in stunted growth and reduced yield and fruit quality. Our previous studies evaluated melon germplasm resources under HTH stress at the seedling stage and identified two contrasting genotypes: the HTH-tolerant cultivar ‘JIN DI’ (JD) and the HTH-sensitive cultivar ‘JIN TAI LANG’ (JTL) [22]. Furthermore, the differences in physiological and photosynthetic responses to HTH stress between the two varieties were analyzed [23]. Nevertheless, the molecular identity and expression dynamics of the *HSP70* family in these two melon genotypes under HTH stress remain unknown. Here, we performed a genome-wide survey of the *CmHSP70* family in melon through comprehensive bioinformatic analyses. Furthermore, by integrating RNA-seq with qRT-PCR, we compared *CmHSP70* expression between the two cultivars (JD and JTL) across 0, 1, 2, and 3 days of HTH exposure, with the goal to identify candidate genes responsive to HTH tolerance, which can be used for future crop breeding studies.

## 2. Materials and Methods

### 2.1. Genome-Wide Identification and Physicochemical Properties of CmHSP70 Gene Family in Melon

Genomic sequence, protein sequence, and gene structure annotation of melon were obtained from the Cucurbit Genomics Database (http://www.cucurbitgenomics.org/organism/18, accessed on 10 March 2026), and the reference genome version was DHL92 (CM3.6.1). We obtained HMM profiles for the conserved HSP70 domain from Pfam (http://pfam.xfam.org/, accessed on 11 March 2026) [24] and they were used to search the melon non-redundant protein set via HMMER v3.3.2 with an E-value cutoff of 1 × 10^−5^. *Arabidopsis* AtHSP70 protein sequences were downloaded from the Arabidopsis Information Resource (TAIR) (https://www.arabidopsis.org/, accessed on 18 March 2026), and BLASTP v2.16.0 was performed against the melon non-redundant protein database with an E-value < 1 × 10^−5^. Sequences obtained from both strategies were merged, redundancies were removed, and *CmHSP70* members were confirmed using the Conserved Domain Database (CDD, https://www.ncbi.nlm.nih.gov/Structure/cdd/cdd.shtml, accessed on 25 March 2026) and Simple Modular Architecture Research Tool (SMART, https://smart.embl.de, accessed on 12 April 2026). Based on domain integrity, all candidates were classified into two categories: proteins harboring complete or near-complete HSP70 domains were regarded as canonical *CmHSP70* members, whereas abnormally short sequences containing only partial HSP70-related domains were retained as truncated/partial HSP70-like proteins, provided they passed the initial HMMER or BLASTP screening criteria. The final *CmHSP70s* obtained were named according to their order on the chromosome. Molecular weight (MW), isoelectric point (pI), and additional physicochemical characteristics were predicted using the ExPASy proteomics server (https://web.expasy.org/protparam/, accessed on 15 April 2026) following standard procedures [25]. Subcellular targeting was predicted for each CmHSP70 using WoLF PSORT (https://wolfpsort.hgc.jp/, accessed on 20 April 2026).

### 2.2. Chromosomal Localization and Phylogenetic Analysis of CmHSP70 Proteins

Chromosomal positions of *CmHSP70 genes* were drawn from GFF files and plotted with TBtools-II v2.096 [26]. For phylogenetic analysis, HSP70 protein sequences from *Arabidopsis*, rice, melon, pumpkin, watermelon, and cucumber were aligned with MAFFT v7.520 [27]. Using this alignment in MEGA11, a Neighbor-Joining phylogeny was constructed, with node reliability assessed through 1000 bootstrap iterations; the resulting tree was subsequently annotated and rendered using iTOL (https://itol.embl.de/, accessed on 25 April 2026) [28].

### 2.3. Conserved Motifs and Gene Structure of CmHSP70 Proteins

MEME (https://meme-suite.org/meme/tools/meme, accessed on 26 April 2026) was run on the 16 CmHSP70 protein sequences to detect conserved motifs, with the search limited to 10 motifs and all other options unchanged [29]. TBtools-II v2.096 was then used to generate a combined figure showing the phylogenetic tree, MEME motif architecture, and exon–intron organization extracted from GFF annotations.

### 2.4. Cis-Acting Element Analysis in CmHSP70 Promoters

Putative promoter sequences were defined as the 2000 bp region immediately upstream of the ATG start codon for each CmHSP70 gene. This upstream region was then screened via the PlantCARE web tool (https://bioinformatics.psb.ugent.be/webtools/plantcare/html/, accessed on 30 April 2026) to annotate putative cis-regulatory elements. They were classified by function into categories such as environmental response, plant hormone response, transcription factor binding, tissue specificity, growth and development, etc. The number distribution of each element in the promoters of different members was statistically presented in a heatmap form.

### 2.5. Gene Duplication and Synteny Analysis

Gene duplication events and synteny between melon and other species were analyzed using the MCScanX module in TBtools-II v2.096 [30]. Segmental/tandem duplications were identified from MCScanX, and interspecific collinearity was used to identify orthologous gene relationships. Ka, Ks, and Ka/Ks were then calculated for all pairwise comparisons among the 16 *CmHSP70* coding sequences using the Simple Ka/Ks Calculator in TBtools (Supplementary Table S1). Pairs with dS = 0 or otherwise unreliable estimates (undefined Ka/Ks values, i.e., NAN) were excluded, yielding 62 paralogous pairs with reliable Ka/Ks estimates. A Ka/Ks value below 1 was interpreted as evidence of purifying selection, value equal to 1 as neutral evolution, and a value exceeding 1 as positive selection. The pairwise Ka, Ks, and Ka/Ks values were plotted as a three-dimensional scatter.

### 2.6. RNA-Seq Expression Analysis of HSP70 Gene Family in Melon

Transcriptomics data of several plant tissues including root, stem (stem_downside), leaf (leaves_9th), male flower (male_flower_anther_DAF0d), female flower (female_flower_DAF0_ovary) and fruit (DAF50d_flesh) of melon were downloaded from Melonet DB (https://melonet-db.dna.naro.go.jp/, accessed on 10 May 2026) to analyze the expression levels of *CmHSP70* genes [31]. The transcriptional expression levels of the *CmHSP70* gene family in melon sensitive cultivar JTL and the tolerant cultivar JD under high-temperature and high-humidity stress for 0, 1, 2, and 3 days were extracted from the NCBI database (https://www.ncbi.nlm.nih.gov/bioproject/PRJNA1434721, accessed on 15 May 2026) as submitted in our previous study. The FPKM values of *CmHSP70* family genes were transformed by log_2_(FPKM + 1) using TBtools software, and a gene expression heatmap was constructed.

### 2.7. 3D Structural Prediction and Protein–Protein Interaction Network Analysis

The amino acid sequences of 16 CmHSP70 proteins were submitted to the SWISS-MODEL server (https://swissmodel.expasy.org/, accessed on 15 May 2026) (Supplementary File S1), which automatically performs sequence alignment and searches for optimal templates, then constructs three-dimensional structural models based on homology modeling. Model quality was evaluated using GMQE and QMEAN values, and amino acid positions from the N-terminus to the C-terminus were annotated using a rainbow color scheme. A predicted protein–protein interaction (PPI) network was constructed using the STRING database (v12.0, https://string-db.org/, accessed on 15 May 2026) with the “Proteins by sequences” function. The amino acid sequences of 16 CmHSP70 proteins were submitted sequentially with *C. melo* as the reference organism and a minimum interaction confidence threshold of 0.400 to obtain interaction relationships and combined confidence scores. STRING associations are predicted (not experimentally verified in melon), and gene-to-node mapping is provided in Supplementary Table S2. The interaction data were imported into Cytoscape software (v3.10.4). Topological parameters including Degree and Betweenness Centrality were calculated using the built-in Network Analyzer plugin (Cytoscape v3.10.4), and network module clustering was performed using the MCODE plugin v2.0.2 (degree cutoff = 2, K-core = 2). Hub genes were defined a priori as nodes with Degree ≥ 10. The network was visualized using the NetworkX v3.6 and Matplotlib v3.11.1 libraries in Python v3.14.7, with node size mapped to Degree values, node color to module assignment, and edge width to interaction confidence scores.

### 2.8. Plant Growth and qRT-PCR Analysis of HSP70 Gene Family

Seedlings of the high temperature and humidity-sensitive cultivar ‘JIN TAI LANG’ (JTL) and tolerant cultivar ‘JIN DI’ (JD) were grown and subjected to combined high temperature and humidity stress as described in our previous study. Briefly, at the three-leaf stage, seedlings were treated at 45 °C/35 °C (day/night, 12 h/12 h) with 85%/95% relative humidity and 100% soil moisture. The second true leaf was sampled at 0, 1, 2, and 3 days after treatment [23]. Total RNA was isolated from melon leaves using the MolPure^®^ Plant RNA Kit (Yeasen, Shanghai, China). Subsequently, 1 μg of qualified total RNA was reverse-transcribed into first-strand cDNA with the Hifair^®^ III 1st Strand cDNA Synthesis SuperMix for qPCR (gDNA digester plus) (Yeasen, Shanghai, China). qRT-PCR amplification was performed on a LightCycler480^®^ II Real-Time System (Roche, Foster City, CA, USA) utilizing Hieff UNICON^®^ ColorGPS qPCR SYBR Green Master Mix (No Rox) (Yeasen, Shanghai, China). The thermal cycling program was configured as an initial denaturation step at 95 °C for 3 min, followed by 40 amplification cycles consisting of 95 °C for 5 s, 60 °C for 10 s, and 72 °C for 15 s. Three biological replicates and three technical replicates were set for each sample to ensure experimental reliability. The amplification efficiency of each primer pair was determined using the standard curve method with a serial dilution of pooled cDNA, ranging from 94.5% to 106.3%, with *R*^2^ > 0.99 (Supplementary Table S3). Melting curve analysis was performed to confirm a single specific amplicon for each primer pair. The 2^−ΔΔCt^ method was applied to calculate relative gene expression, using Actin as the internal reference gene [32]. The stability of Actin was assessed separately within each cultivar by comparing its Ct values across time points using one-way ANOVA, and no significant difference was detected in either JD or JTL (*p* > 0.05), confirming its suitability as a reference gene under HTH stress. For each cultivar, the sample at day 0 under normal growth conditions was used as the calibrator (relative expression set to 1), except for *CmHSP70-7* and *CmHSP70-8*, whose transcripts were undetectable at day 0 in both cultivars; for these two genes, the day 1 sample was used as the calibrator instead, and day 0 data points were excluded from the statistical analysis. The sequences of primers used are presented in Supplementary Table S3.

### 2.9. Statistical Analysis

GraphPad Prism 11.0.1 (GraphPad Software, San Diego, CA, USA) was used for statistical analysis. Two-way ANOVA was used to examine the effects of cultivar, treatment time, and their interaction on qRT-PCR expression data, and Tukey’s test was subsequently applied for pairwise comparisons within each cultivar. Differences were considered significant at *p* < 0.05. For the transcriptome data, log_2_ (FPKM + 1)-transformed values were used for heatmap visualization, and the expression data were statistically evaluated as follows: genes with a maximum FPKM below 1 across all samples (*CmHSP70-1*, *-9*, and *-16*) were considered not expressed and excluded, and the log_2_ (FPKM + 1) values of the remaining 13 *CmHSP70* genes were subjected to two-way ANOVA to test the effects of cultivar, treatment time, and their interaction (*n* = 3), with the full results provided in Supplementary Table S4.

## 3. Results

### 3.1. Genome-Wide Characterization of CmHSP70 Members and Their Physicochemical Features in Melon

A genome-wide search identified 16 *CmHSP70* genes in melon, numbered *CmHSP70-1* to *CmHSP70-16* by chromosomal position, with their physicochemical parameters and predicted localizations listed in Table 1. Protein lengths ranged from 94 to 898 aa (10.26–99.93 kDa). Most members are full-length or near full-length (~600–900 aa). *CmHSP70-1* (121 aa), *CmHSP70-3* (201 aa), and *CmHSP70-9* (94 aa) are markedly shorter and retain only partial HSP70-related domains (Supplementary Table S5); they were classified as truncated/partial HSP70-like proteins (possibly incomplete models, truncated annotations, or HSP70-related fragments). *CmHSP70-16* (326 aa) is an HSP70-related chimeric protein (short ASKHA_ATPase-like region plus DUF4283). All 16 loci were retained to survey HSP70-related sequences in the current annotation. Their theoretical isoelectric points (pI) are distributed between 5.03 and 9.71, with 14 of 16 (87.5%) members being acidic proteins (pI < 7) and only *CmHSP70-1* (pI = 7.77) and *CmHSP70-16* (pI = 9.71) being basic proteins. Instability index (II) values spanned 26.46–46.35 across the family; *CmHSP70-4*, *CmHSP70-5*, and *CmHSP70-10* exceeded the 40 threshold and were thus classified as unstable, whereas all other members fell into the stable category. The aliphatic index (AI) ranges from 70.91 to 103.72, indicating that the CmHSP70 family proteins as a whole exhibit good thermal stability. The analysis of protein hydrophilicity and hydrophobicity revealed that only *CmHSP70-8* and *CmHSP70-9* exhibited a GRAVY value greater than 0, indicating that they are hydrophobic proteins. Conversely, all other members had a GRAVY value less than 0, suggesting they are hydrophilic proteins. The subcellular localization prediction indicated that the members of the *CmHSP70* gene family exhibit diverse intracellular distributions. Most of the members are predicted to be located in the cytoplasm, four members are predicted to be found in chloroplasts, and two members are predicted to be located in the endoplasmic reticulum, with *CmHSP70-1* predicted to localize in the extracellular region and *CmHSP70-5* predicted to localize in the nucleus. These computational predictions suggest that different *CmHSP70* family members might exert their molecular chaperone function in various cellular compartments.

### 3.2. Chromosomal Localization and Protein Phylogeny of CmHSP70 Genes in Melon

**Figure 1:**
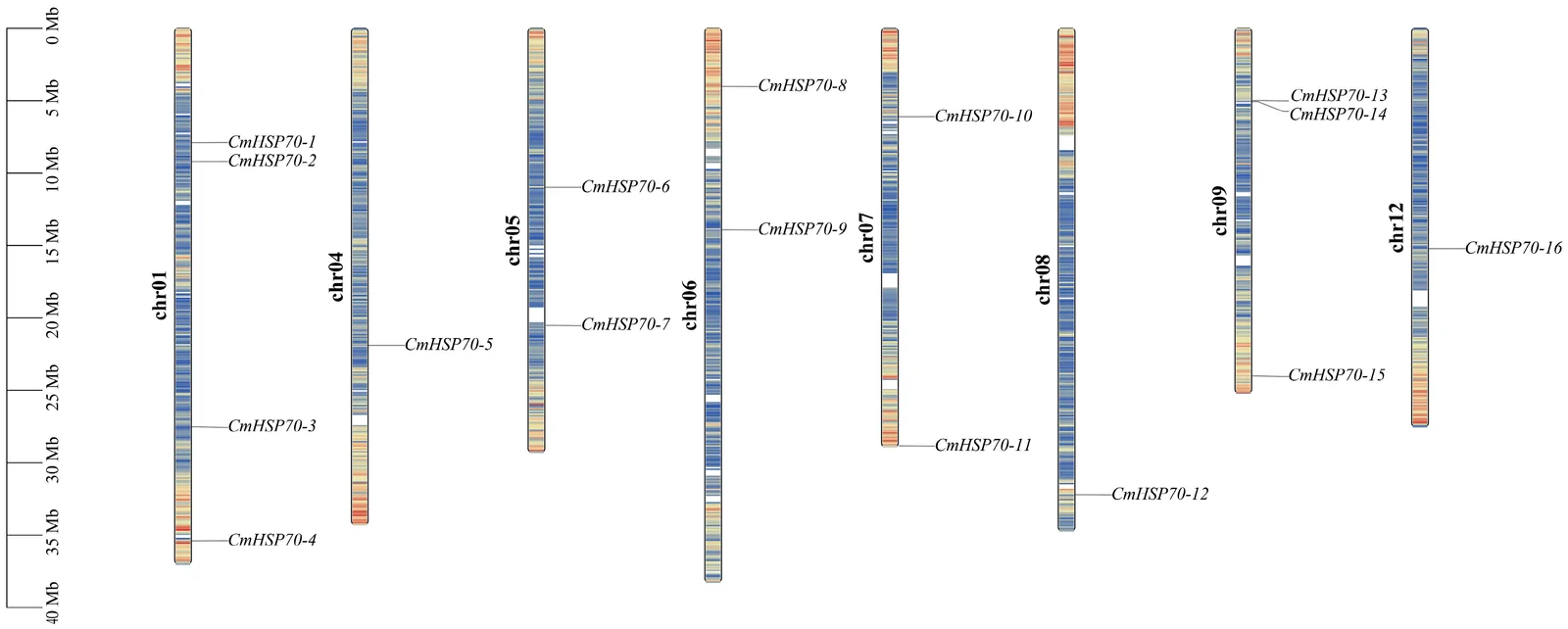
Chromosomal mapping of 16 *CmHSP70* genes in the melon genome. Numbers at the top of each bar denote chromosome identifiers. The left-side scale bar represents physical distance in megabases (Mb).

Chromosomal localization of the 16 identified *CmHSP70* genes in melon was performed using TBtools, based on the melon whole-genome sequence downloaded from the database (Figure 1). Our findings showed that the *CmHSP70* family genes are unevenly distributed across eight of the twelve melon chromosomes, exhibiting a significant imbalance in their overall distribution. Chromosome 1 carries the largest share of *CmHSP70* members, *CmHSP70-1*, *CmHSP70-2*, *CmHSP70-3*, and *CmHSP70-4*, making it the primary chromosomal hub for this gene family. Next is chromosome 9, which has three gene members: *CmHSP70-13*, *CmHSP70-14*, and *CmHSP70-15*. Chromosomes 5, 6, and 7 each contain two *CmHSP70* genes; one *CmHSP70* family gene is located on chromosomes 4, 8, and 12, respectively. In addition, some *CmHSP70* genes on certain chromosomes exhibit a clustered distribution pattern, suggesting that local gene duplication may have shaped this family’s evolutionary history that likely underlies the functional divergence and varied stress-response behavior seen across the *CmHSP70* family.

**Figure 2:**
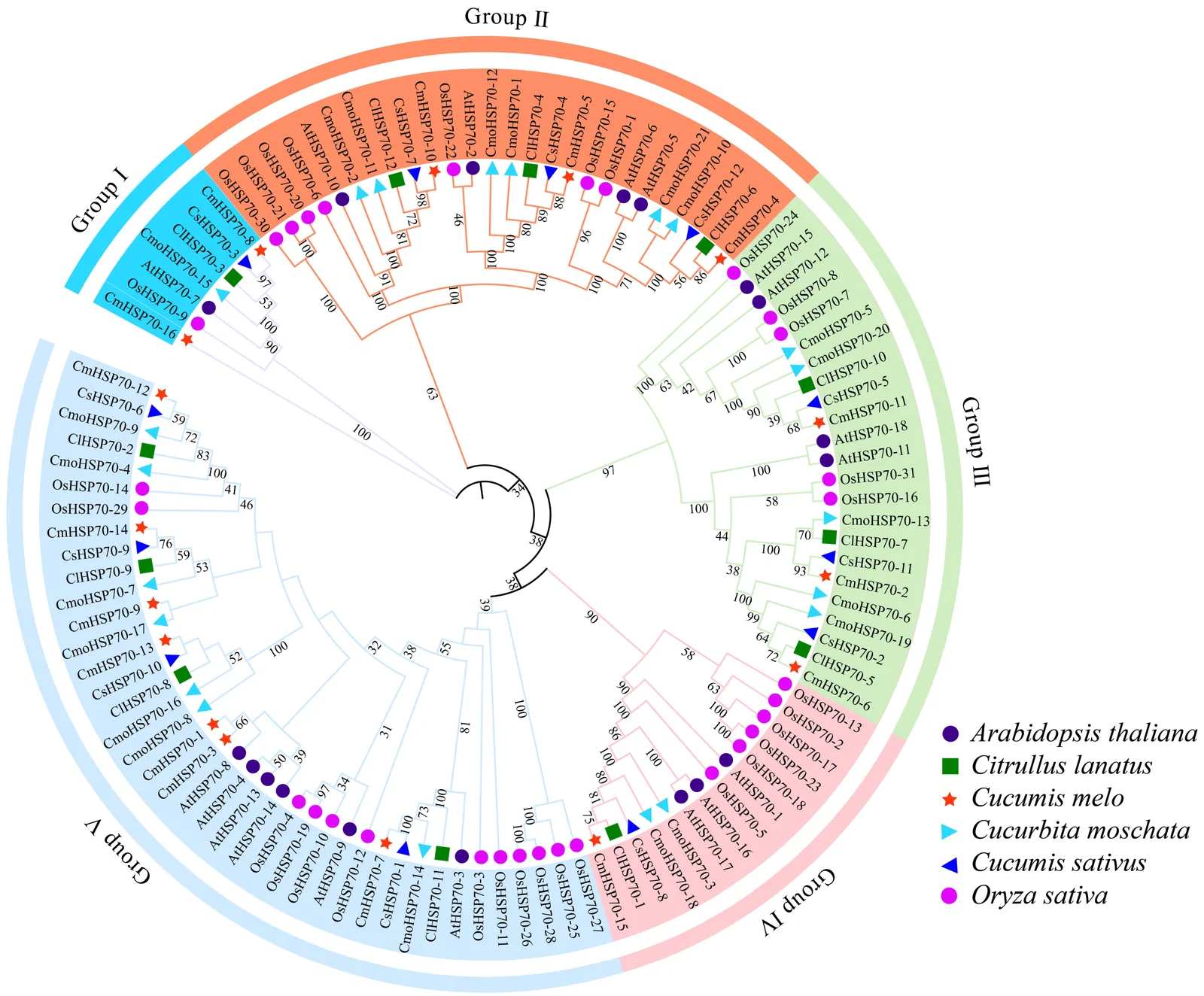
Phylogenetic relationship of HSP70 proteins from melon and five other plant species. HSP70 proteins from *Citrullus lanatus* (Cl), *Oryza sativa* (Os), *Arabidopsis thaliana* (At), *Cucurbita moschata* (Cmo), *Cucumis sativus* (Cs), and *Cucumis melo* (Cm) are included. The five subfamilies are designated Groups I–V.

To clarify the phylogenetic relationships of the *CmHSP70* family among different species, a phylogenetic tree of HSP70 proteins from *Oryza sativa*, *Arabidopsis thaliana*, *Citrullus lanatus*, *Cucurbita moschata*, *Cucumis sativus*, and *Cucumis melo* (melon) was constructed using MEGA11 software (Figure 2). The results revealed that the 16 *HSP70* proteins in melon could be clearly divided into five subfamilies: Group I contains two HSP70 proteins, Group II contains three HSP70 proteins, Group III contains three HSP70 proteins, and Group IV contains one HSP70 protein. Group V is the largest dominant subgroup and the main enrichment branch of melon CmHSP70 protein, consisting of seven HSP70 proteins. The vast majority of *CmHSP70* family members are concentrated in Group V and closely clustered with HSP70 proteins in cucurbitaceae crops such as watermelon, cucumber, and pumpkin. They have the closest genetic relationship and the strongest sequence conservation. In general, the HSP70 protein in melon is highly consistent with the evolutionary relationship of cucurbitaceae species, while exhibiting significant species-specific evolutionary differentiation with *A. thaliana* and *O. sativa*.

### 3.3. Conserved Motifs and Gene Structure of CmHSP70 Gene Family

**Figure 3:**
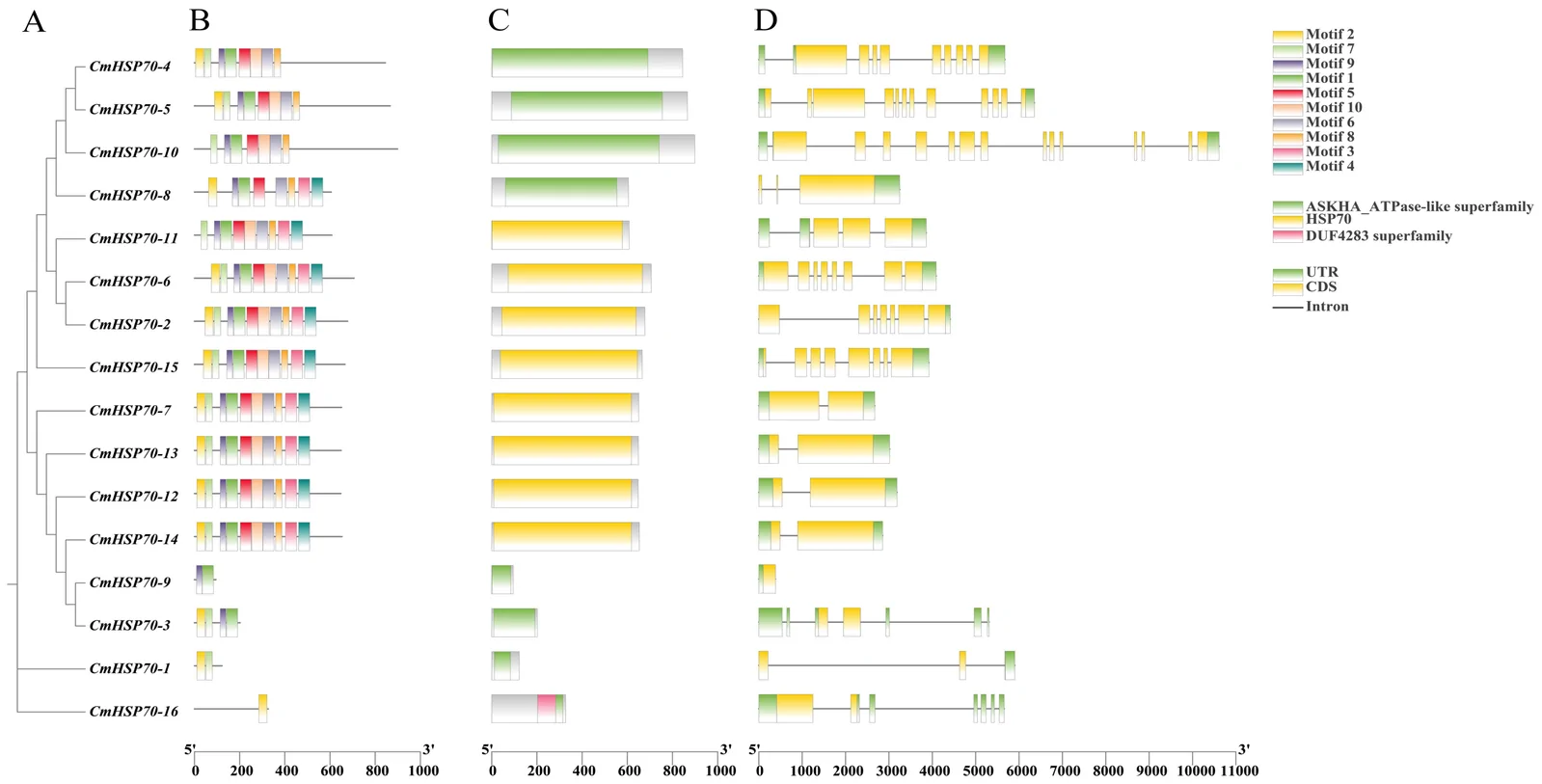
Genomic structure and conserved motifs of the CmHSP70 gene family. (**A**) Phylogenetic tree of *CmHSP70* proteins. (**B**) Distribution of ten conserved motifs. (**C**) Conserved domain architecture of CmHSP70 proteins predicted by CDD and SMART, with gray shading representing regions lacking conserved domains. (**D**) Exon–intron structure of *CmHSP70* genes.

The MEME tool was applied to screen for conserved motifs across the *CmHSP70* gene family, from which 10 distinct motifs were identified (Figure 3). Among them, seven *CmHSP70* genes contain 10 conserved motifs, and most *CmHSP70* genes have more than seven conserved motifs. However, a few members have obvious motif deletions, such as *CmHSP70-1*, *CmHSP70-3*, *CmHSP70-9*, and *CmHSP70-16*, which only contain one to four conserved motifs and lack some specific motifs, showing significant structural specificity (Figure 3B). Most CmHSP70 proteins contain ASKHA-ATPase-like or intact HSP70 domains. Consistent with their short proteins, *CmHSP70-1*, *CmHSP70-3*, and *CmHSP70-9* show extensive motif/domain loss and are best interpreted as truncated/partial HSP70-like sequences rather than complete canonical HSP70s (Figure 3C; Supplementary Table S5). In addition, *CmHSP70-16* has the farthest evolutionary distance and exhibits a unique chimeric domain architecture: it retains a short N-terminal ASKHA_ATPase-like domain but lacks the canonical C-terminal HSP70 substrate-binding domain, which is replaced by a DUF4283 superfamily domain (Figure 3C). The analysis of the exon–intron structure of the *CmHSP70* family genes showed significant differences in structural differentiation, with a variation range of 0–14 intron numbers. The genetic structural characteristics of different members vary significantly, with only one intron in genes such as *CmHSP70-7*, *CmHSP70-12*, *CmHSP70-13*, and *CmHSP70-14*, resulting in a simplified gene structure and compact exon arrangement. In contrast, *CmHSP70-5* and *CmHSP70-10* genes have longer sequences and more complex structures (Figure 3D). The above results indicate that *CmHSP70* members within the same evolutionary subgroup have highly similar exon numbers, intron distribution patterns, and gene lengths.

### 3.4. Analysis of Cis-Acting Elements in the Promoters of CmHSP70 Gene Family

**Figure 4:**
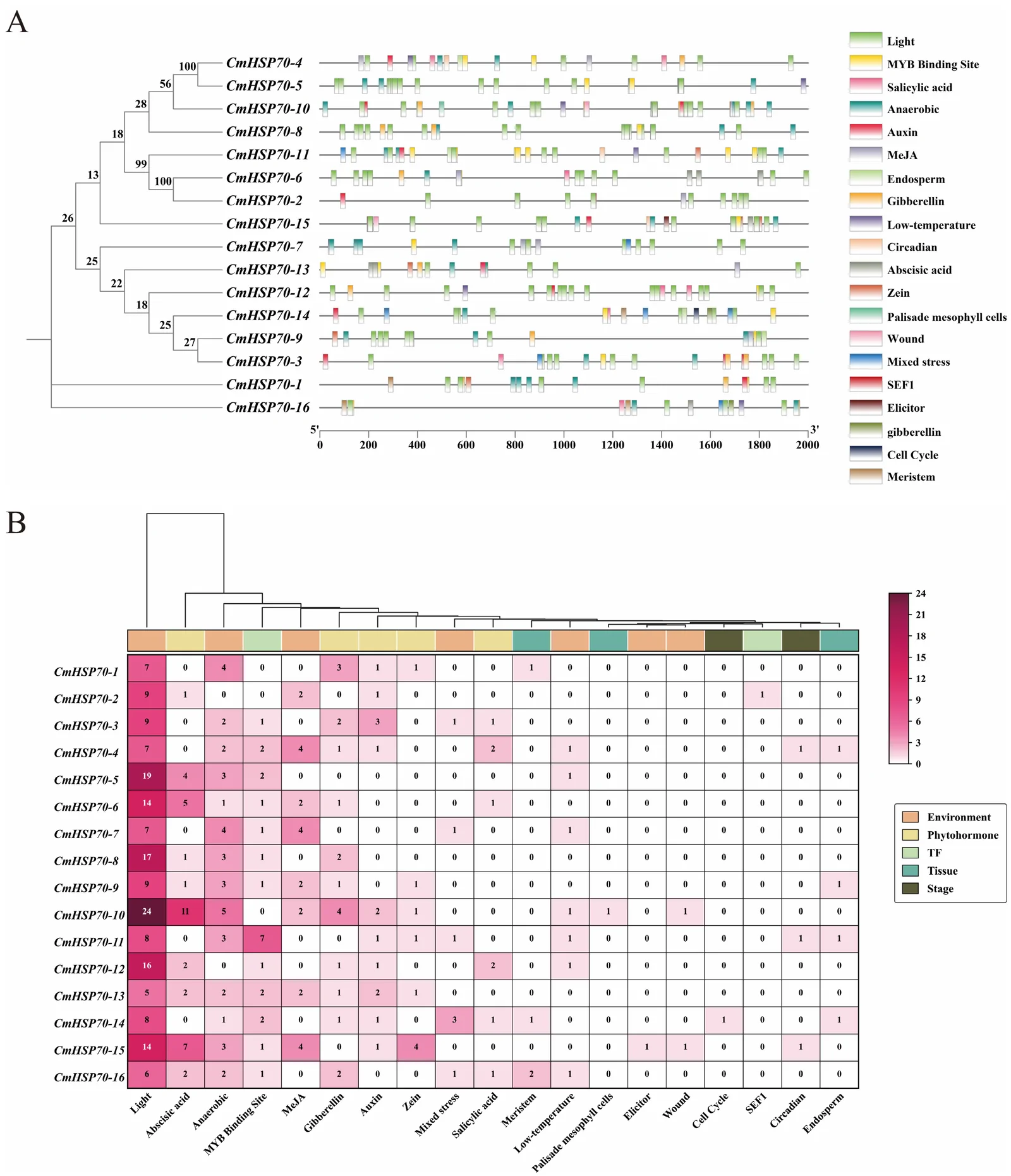
Analysis of cis-acting elements in *CmHSP70* gene promoters of melon. (**A**) Distribution of predicted cis-acting elements along the promoter regions of *CmHSP70* genes and (**B**) heatmap illustrating the abundance of each cis-acting element across *CmHSP70* promoters.

To characterize the potential transcriptional regulation of *CmHSP70* genes, cis-acting elements were predicted within the 2000 bp promoter regions upstream of all 16 identified *CmHSP70* members in melon. Their distribution patterns across individual promoters are visualized in Figure 4A, while the abundance of each element type is displayed as a heatmap in Figure 4B. Every *CmHSP70* promoter carried regulatory elements linked to light signaling, hormone and stress responses, and tissue-specific development, though family members differed substantially in element type, copy number, and spatial arrangement (Figure 4A). Light-responsive elements are widely distributed in all CmHSP70 promoters, with *CmHSP70-10* having the highest number of 24 light-responsive elements and forming an element enrichment region in the middle of the promoter (Figure 4B). Hormone responsive elements such as MeJA, Salicylic acid, Abscisic acid, Auxin, and Gibberellin are mainly enriched in *CmHSP70-6*, *CmHSP70-10*, and *CmHSP70-15* (each containing 9–15 hormone-related elements), while *CmHSP70-3*, *CmHSP70-4*, and *CmHSP70-5* also harbor a moderate number of such elements, pointing to a role for these genes in hormone-driven responses to abiotic stress (Figure 4B). Stress-related elements including Anaerobic, Low-temperature, MYB Binding Site, Wound, and Mixed stress are also detected in multiple *CmHSP70* promoters. Developmental-specific elements such as Meristem, Endosperm, and Cell Cycle are only distributed in a few members. In addition, Circadian, Zein, Palisade mesophyll cells, SEF1, and Elicitor elements are found in individual *CmHSP70* promoters. These predicted cis-element patterns suggest that the promoter sequences of the *CmHSP70* family in melon may have undergone divergent evolution. Such differences in the types, quantities, and spatial arrangements of cis-elements raise hypotheses about how these family members respond to external signals and acquire functional differentiation.

### 3.5. Synteny, Gene Duplication, and Ka/Ks Selection Pressure of CmHSP70 Genes

**Figure 5:**
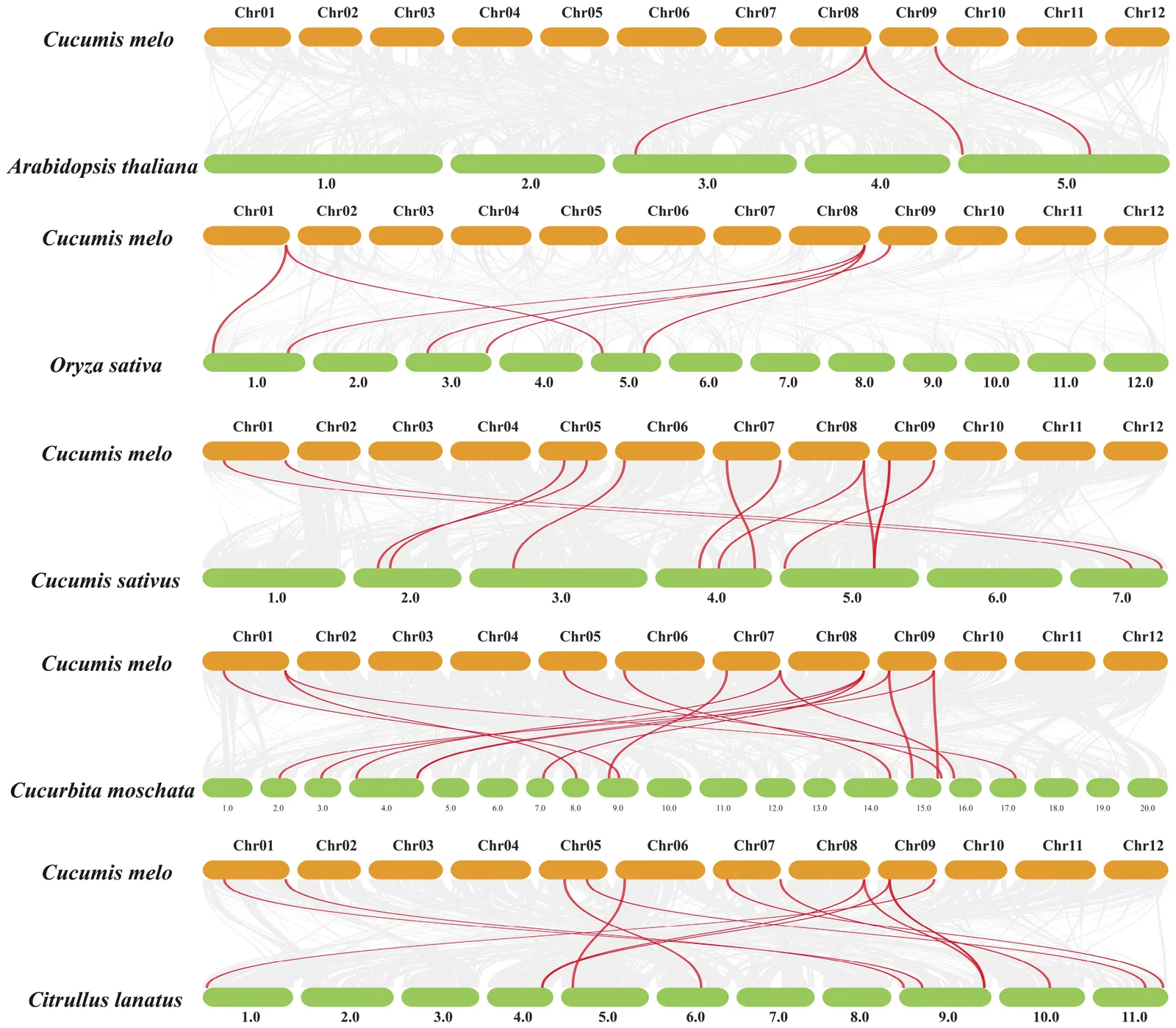
Synteny analysis of the *HSP70* gene family between melon and other species.

To better understand the evolutionary relationships of the *HSP70* gene family across species, synteny analysis was performed between the *HSP70* genes of melon (*Cucumis melo*) and those of *Arabidopsis* (*Arabidopsis thaliana*), rice (*Oryza sativa*.), watermelon (*Citrullus lanatus*), pumpkin (*Cucurbita moschata*), and cucumber (*Cucumis sativus*) (Figure 5). Among the species compared, melon *CmHSP70* genes shared the greatest number of collinear pairs with *C. moschata* (14 pairs), followed by *C. lanatus* (12 pairs) and *C. sativus* (11 pairs). However, there are fewer collinear gene pairs with *A. thaliana* and *O. sativa*, with a total of three and six, respectively. This synteny analysis helps to reveal the conservation and specificity of the *HSP70* gene family during the evolutionary process.

**Figure 6:**
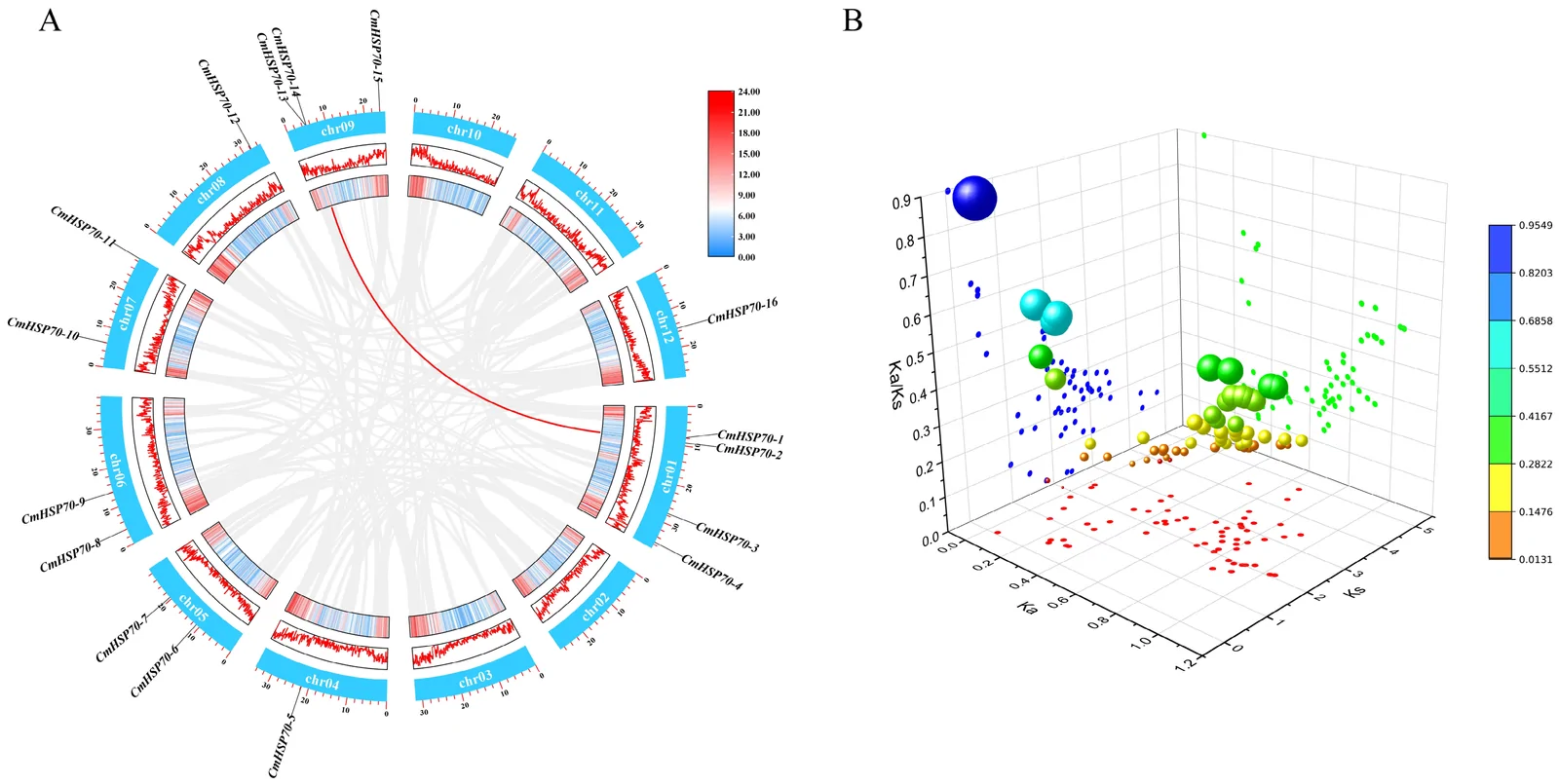
Intra-species synteny and Ka/Ks analysis of *HSP70* genes. (**A**) Intra-species synteny; the MCScanX segmental pair CmHSP70-2/CmHSP70-14 is indicated. (**B**) Ka, Ks, and Ka/Ks for the 62 paralogous *CmHSP70* pairs with reliable estimates.

Intra-species collinearity analysis identified only one MCScanX-supported segmental duplication pair, *CmHSP70-2* and *CmHSP70-14* (Figure 6A), and no tandem clusters, indicating limited recent expansion via segmental/tandem duplication. Separately, Ka/Ks values were calculated for paralogous *CmHSP70* pairs with reliable estimates (62 pairs; Supplementary Table S1 and Figure 6B). All Ka/Ks ratios were <1, indicating purifying selection and high functional conservation of the encoded proteins. The segmental duplication pair *CmHSP70-2*/*CmHSP70-14* had Ka/Ks = 0.202. The numerous points in Figure 6B correspond to these 62 pairwise comparisons, not only the single segmental pair in Figure 6A. Taken together, these results indicate that only a limited subset of the homologous genes originating from ancestral whole-genome duplication has persisted in the *CmHSP70* family. Following duplication, the coding sequences have maintained conserved structures under purifying selection, whereas sequence variations in the regulatory regions have driven subfunctionalization of the homologous genes, ultimately giving rise to the diverse stress-response profiles observed among the current *CmHSP70* family members.

### 3.6. Expression Analysis of Melon HSP70 Genes in Different Tissues

**Figure 7:**
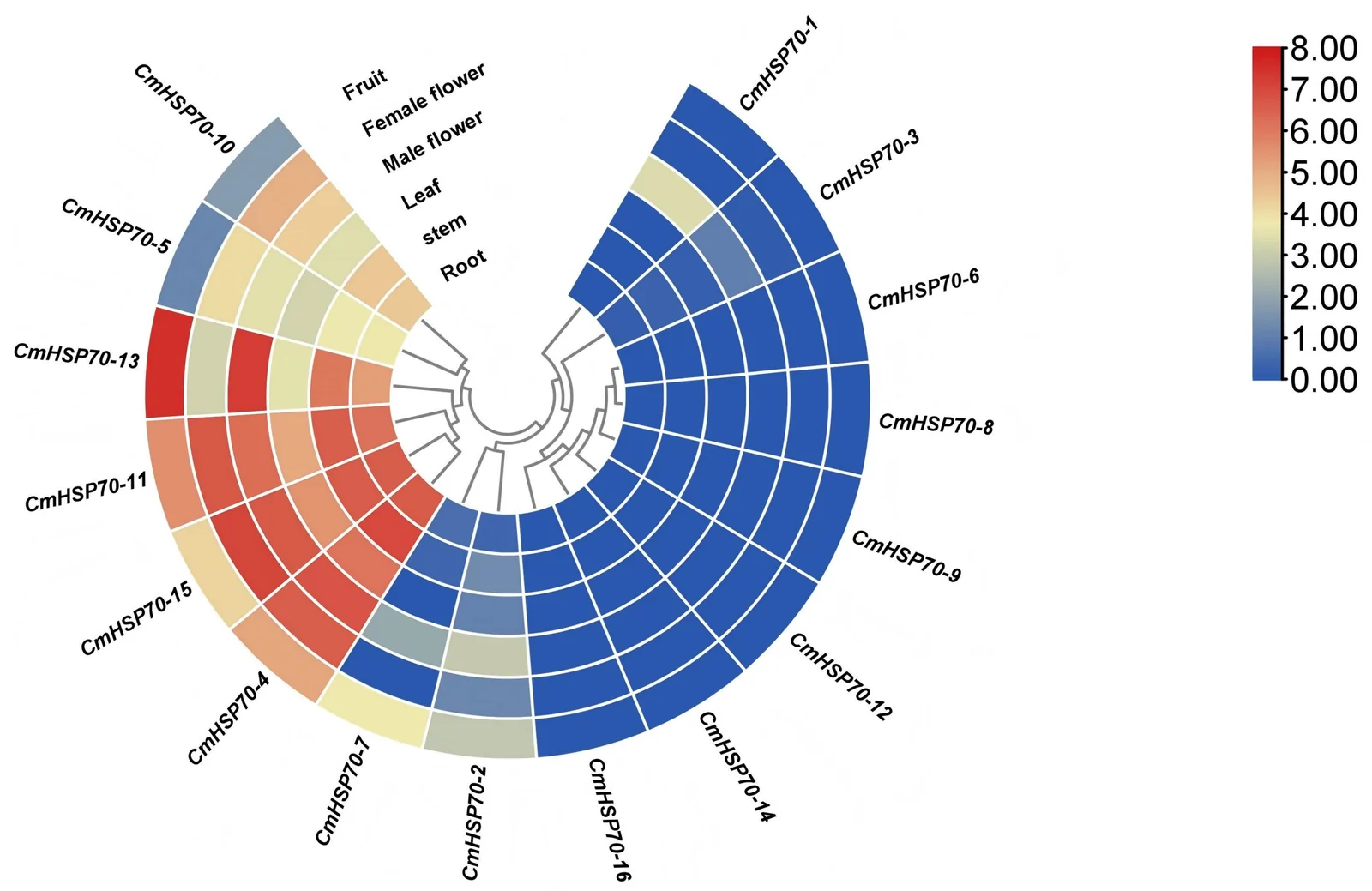
Tissue-specific expression profiles of *CmHSP70* genes in melon. The color gradient from blue to red represents log_2_ (FPKM + 1)-transformed expression values, with the scale bar indicating the magnitude of gene expression.

This heatmap illustrates the expression profiles of 16 *CmHSP70* genes across various melon tissues, revealing pronounced expression divergence within the gene family (Figure 7). Among them, *CmHSP70-4*, *CmHSP70-11*, and *CmHSP70-15* maintain consistently high expression levels across all tissues examined, representing constitutively expressed members that likely serve fundamental molecular chaperone and heat-shock protective functions throughout melon growth and development. In contrast, *CmHSP70-13* exhibits broad expression with the highest levels in fruit, male flowers, and leaves, and moderate levels in stems and roots, suggesting its potential involvement in both vegetative and reproductive organ development. *CmHSP70-5* and *CmHSP70-10* show moderate expression in most tissues but are downregulated in fruit. Among the remaining members, seven genes (*CmHSP70-1/6/8/9/12/14/16*) displayed negligible or undetectable expression across all six tissues, while *CmHSP70-2*, *CmHSP70-3*, and *CmHSP70-7* showed tissue-specific expression with low-to-moderate levels in specific tissues (e.g., stem, leaf, or fruit) but near-zero expression in the remaining tissues, implying that these members may be stress-inducible or tissue-specifically regulated. Collectively, the divergent expression patterns between vegetative and reproductive organs, together with the co-existence of constitutive and tissue-specific members, reflect the functional diversity and division of labor within the melon *HSP70* gene family.

### 3.7. Expression of CmHSP70 Genes Under High Temperature and Humidity Stress in Melon

**Figure 8:**
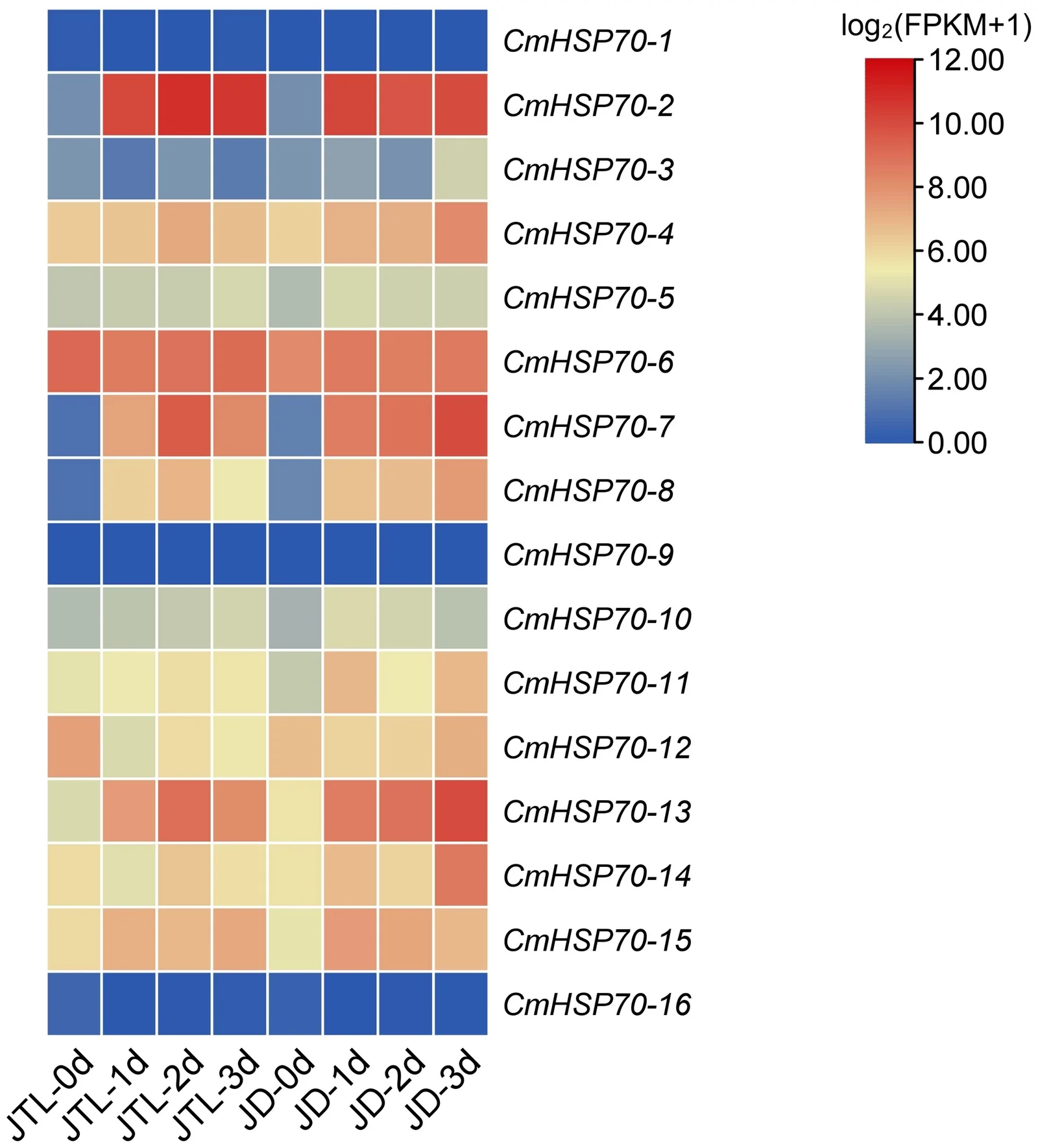
Expression profiles of *CmHSP70* genes in melon leaf tissues under high temperature and humidity (HTH) stress. Transcriptome data were obtained from two melon cultivars, the HTH-sensitive ‘JIN TAI LANG’ (JTL) and the HTH-tolerant ‘JIN DI’ (JD), at 0, 1, 2, and 3 days after HTH treatment. FPKM values were transformed by log_2_(FPKM + 1). The color gradient from blue (low) to red (high) indicates log_2_(FPKM + 1) expression values (scale: 0–12). Expression differences were statistically evaluated by two-way ANOVA on the 13 assessable genes, with *CmHSP70-1*, *-9*, and *-16* excluded as not expressed (maximum FPKM < 1 across all samples) (cultivar × time; *n* = 3).

To investigate the transcriptional response of the *CmHSP70* gene family to combined high temperature and humidity (HTH) stress, transcriptome data were used to compare the expression dynamics of 16 *CmHSP70* members in the sensitive cultivar JTL and the tolerant cultivar JD at 0, 1, 2, and 3 days of HTH treatment (Figure 8). Three genes, *CmHSP70-1*, *CmHSP70-9*, and *CmHSP70-16*, remained at extremely low or undetectable expression levels across all time points in both cultivars and were not induced by HTH stress. *CmHSP70-3*, *CmHSP70-5*, *CmHSP70-10*, and *CmHSP70-11* showed constitutive expression with limited induction. *CmHSP70-6* and *CmHSP70-12* exhibited high basal expression at day 0 but responded differently to HTH stress: *CmHSP70-6* maintained high expression throughout stress in both cultivars with higher basal levels in JTL than in JD, whereas *CmHSP70-12* was sharply suppressed at day 1 in JTL before partial recovery and declined initially then recovered by day 3 in JD.

The remaining seven genes, *CmHSP70-2*, *CmHSP70-4*, *CmHSP70-7*, *CmHSP70-8*, *CmHSP70-13*, *CmHSP70-14*, and *CmHSP70-15*, were strongly induced by HTH stress, with contrasting temporal dynamics between the two cultivars. *CmHSP70-2* was rapidly and strongly induced from day 1 in both cultivars and maintained high expression through day 3, with overall higher levels in JTL than in JD. In contrast, *CmHSP70-4* showed sustained upregulation through day 3 in JD but peaked at day 2 then declined in JTL. *CmHSP70-7* followed a similar pattern but with far stronger induction, continuing to rise through day 3 in JD while declining markedly after day 2 in JTL. *CmHSP70-8* also accumulated steadily in JD but showed transient induction followed by decline in JTL. Likewise, *CmHSP70-13* was among the most strongly induced members, sustaining high expression through day 3 in JD but peaking at day 2 then declining in JTL. *CmHSP70-14* was sharply induced at day 3 in JD while remaining low and fluctuating in JTL. *CmHSP70-15* peaked at day 1 in JD then declined, whereas it showed a gradual increase in JTL. Together, the sustained high expression of *CmHSP70-4*, *CmHSP70-7*, *CmHSP70-8*, and *CmHSP70-13* in JD, in contrast to their transient induction followed by decline in JTL, is associated with the contrasting HTH responses of these two cultivars. Because only one tolerant and one sensitive cultivar were compared, these differences indicate association rather than causation. Thus, *CmHSP70-4*, *-7*, *-8*, and *-13* are priority candidates for functional validation. Consistent with these expression patterns, two-way ANOVA confirmed significant cultivar and time effects for *CmHSP70-4*, *-7*, *-8*, and *-13* (*p* < 0.05), and a significant cultivar × time interaction for *CmHSP70-4* (*p* = 0.004), *CmHSP70-8* (*p* = 0.004), and *CmHSP70-13* (*p* = 0.003), whereas the interaction for CmHSP70-7 was marginally non-significant (*p* = 0.091; Supplementary Table S4).

### 3.8. 3D Structure Prediction and Interaction Network Analysis of CmHSP70 Proteins

**Figure 9:**
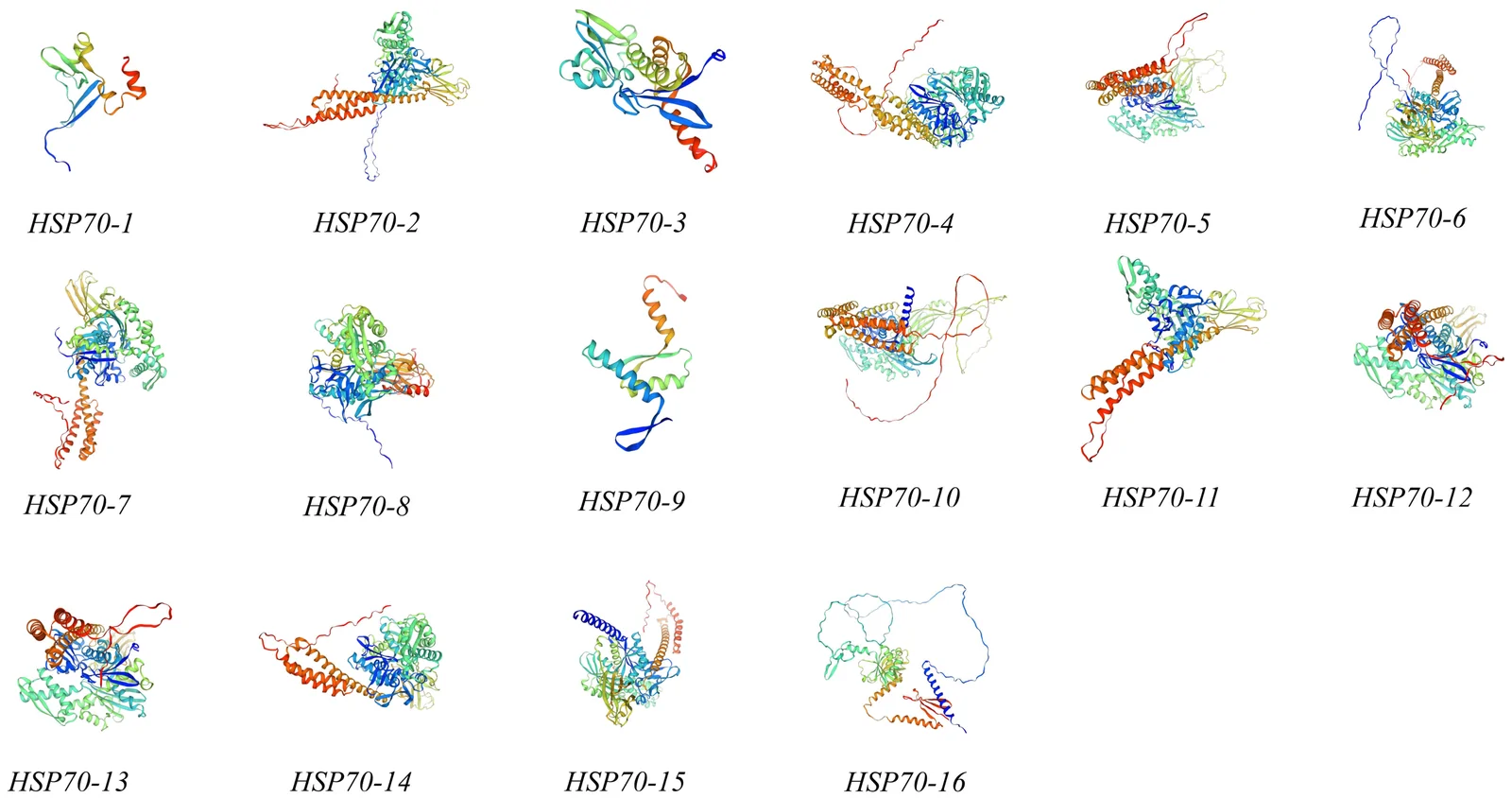
Predicted three-dimensional structures of 16 CmHSP70 proteins in melon. Three-dimensional structures were predicted using SWISS-MODEL. The color gradient from blue to red represents amino acid positions from the N-terminus to the C-terminus.

To explore the predicted structural features and putative interaction relationships of CmHSP70 members, homology modeling and STRING-based network analysis were performed. Homology modeling using SWISS-MODEL predicted that the majority of 16 CmHSP70 proteins possess the typical dual-domain architecture of the *HSP70* family, consisting of an N-terminal nucleotide-binding domain (NBD) and a C-terminal substrate-binding domain (SBD), with *CmHSP70-16* exhibiting a chimeric arrangement in which the C-terminal SBD is replaced by DUF4283 (Figure 9). Additionally, *CmHSP70-9* and *CmHSP70-16* exhibited extended flexible regions at their N- or C-termini, with *CmHSP70-16*’s extended C-terminal region corresponding to its DUF4283 domain, distinguishing them from other family members and suggesting that these members may possess unique substrate recognition or functional regulatory properties.

**Figure 10:**
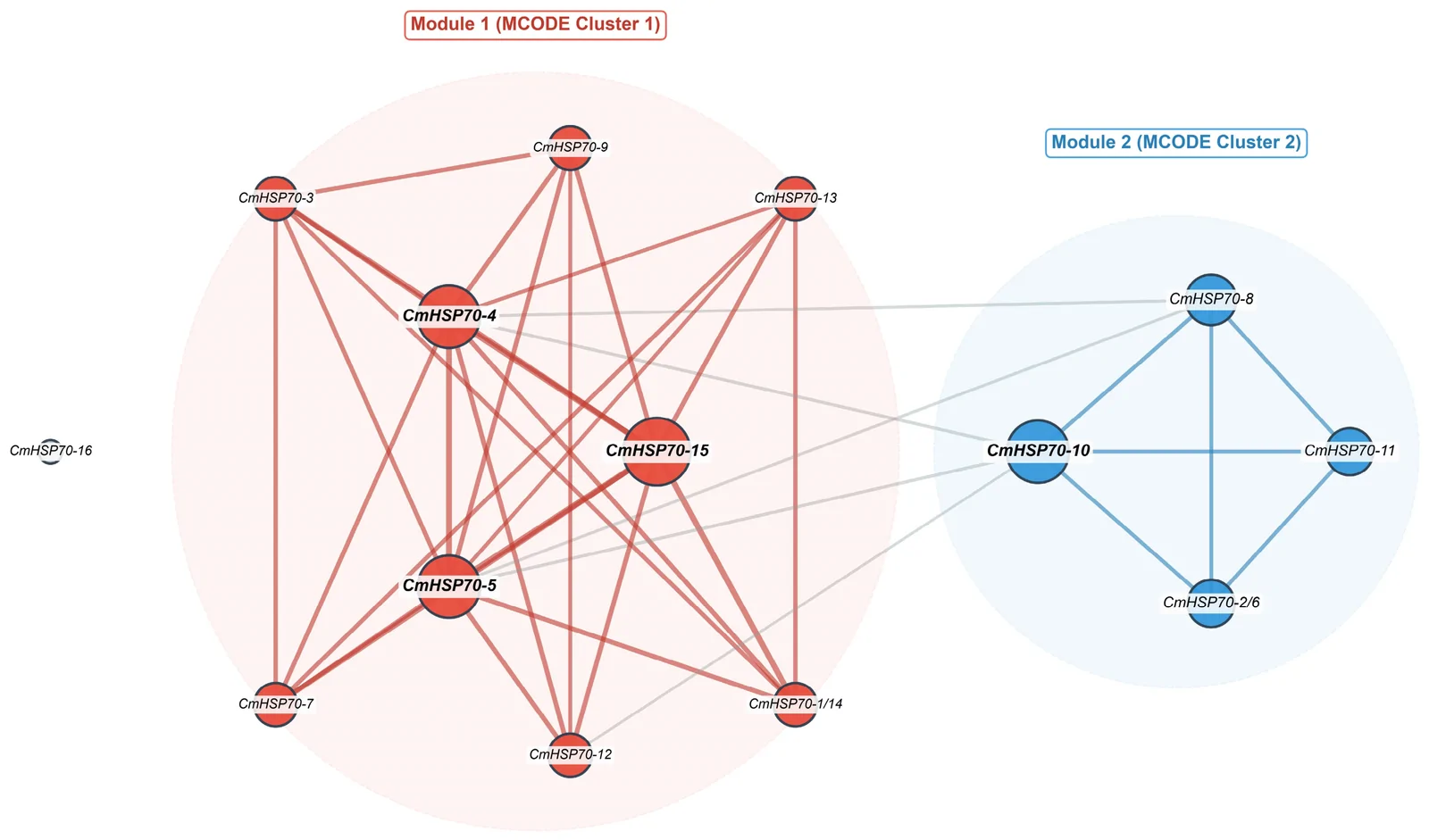
Predicted STRING-based protein–protein interaction network of CmHSP70 proteins in melon. The network was constructed with a confidence threshold of ≥0.400. Node size is proportional to Degree, node color indicates MCODE module assignment (red: Module 1; blue: Module 2; gray: isolated CmHSP70-16), and edge width corresponds to combined interaction confidence. Owing to high sequence similarity, CmHSP70-1/14 and CmHSP70-2/6 are mapped to shared network nodes. Note that all interactions are predicted rather than experimentally verified in melon.

The predicted STRING interaction network contained 14 nodes and 41 edges (Figure 10). Owing to high sequence similarity, CmHSP70-1 and CmHSP70-14 mapped to the same STRING node, as did CmHSP70-2 and CmHSP70-6, limiting gene-specific interpretation for merged nodes (Supplementary Table S2). MCODE clustering analysis partitioned the network into two distinct functional modules: Module 1 comprised nine nodes, corresponding to 10 CmHSP70 proteins (CmHSP70-1/14, CmHSP70-3, CmHSP70-4, CmHSP70-5, CmHSP70-7, CmHSP70-9, CmHSP70-12, CmHSP70-13, CmHSP70-15), with dense intra-module interactions constituting the core region of the network. Module 2 contained four nodes, corresponding to five CmHSP70 proteins (CmHSP70-2/6, CmHSP70-8, CmHSP70-10, CmHSP70-11), forming a relatively independent subnetwork. CmHSP70-16 was identified as an isolated node with no interactions with other family members. Using Degree ≥ 10 as the threshold, a total of four hub genes were identified: CmHSP70-4, CmHSP70-5, CmHSP70-10, and CmHSP70-15, among which CmHSP70-15 exhibited the highest degree (Degree = 12) and occupied the core hub position of the network. Among the four key genes identified in the expression analysis, *CmHSP70-4*, *CmHSP70-7*, and *CmHSP70-13* belonged to Module 1 (with *CmHSP70-4* itself being a hub gene), while *CmHSP70-8* belonged to Module 2. These four key genes were distributed across both functional modules rather than forming an isolated stress-specific cluster in this predicted interaction network, suggesting that the HTH stress response may be integrated into the chaperone interaction network.

### 3.9. qRT-PCR Verification of Gene Expression Levels of the HSP70 Gene Family in Melon Under High Temperature and Humidity Stress

**Figure 11:**
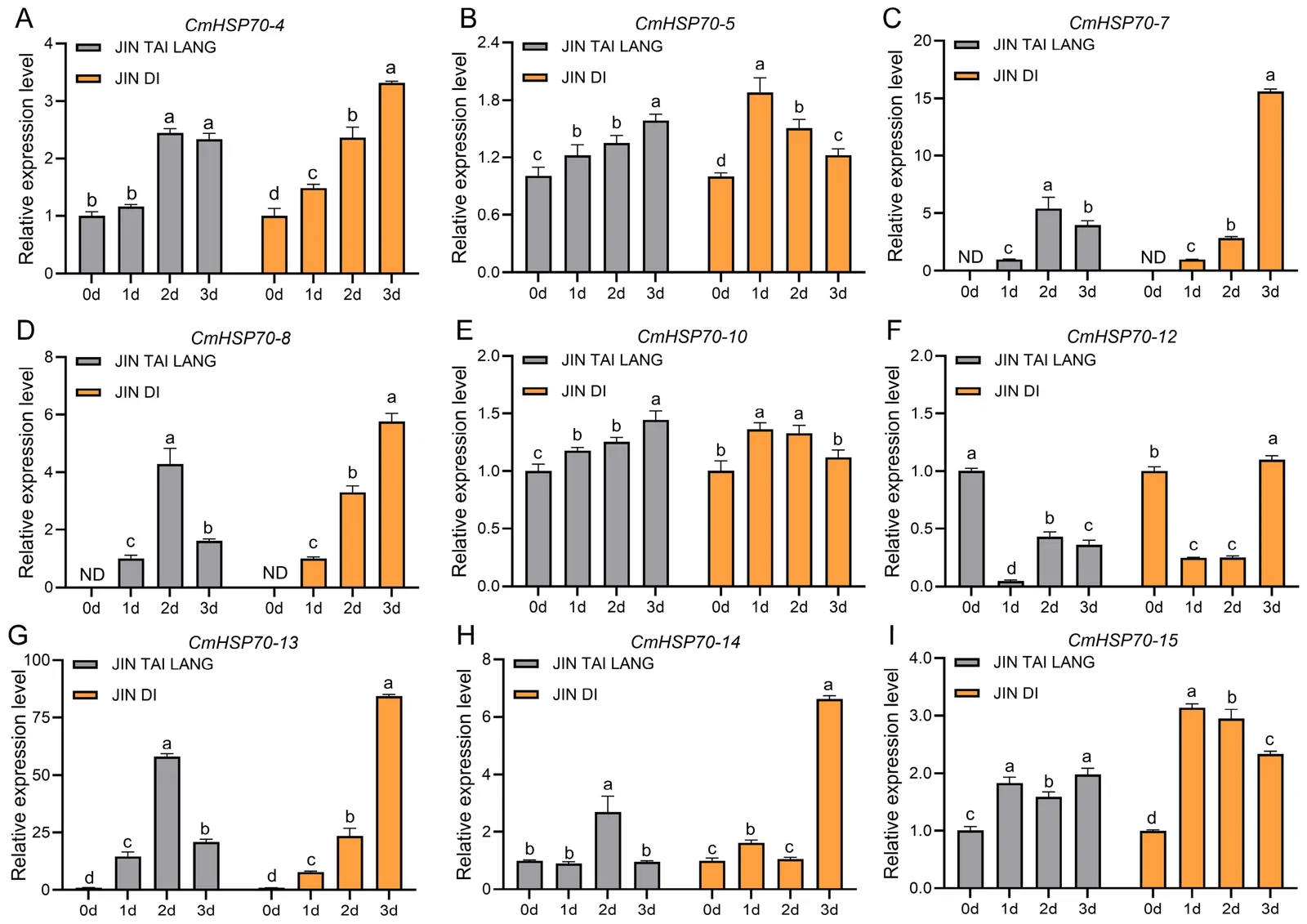
qRT-PCR validation of the expression patterns of nine *CmHSP70* genes under HTH stress in two melon cultivars. The relative expression levels of nine *CmHSP70* genes were determined in seedlings of JTL and JD under 0, 1, 2, and 3 days of HTH stress treatment. Data are means of three biological and technical replicates (*n* = 3). Lowercase letters represent significant differences among treatment time points within each cultivar based on two-way ANOVA followed by Tukey’s test (*p* < 0.05). For *CmHSP70-7* and *CmHSP70-8*, expression was not detected (ND) at day 0; day 1 was used as the calibrator, and day 0 data were excluded from statistical analysis.

To verify the expression characteristics of the *CmHSP70* family under HTH stress, nine genes representing different expression patterns were selected for qRT-PCR validation in two melon cultivars (JTL and JD) (Figure 11). Among them, *CmHSP70-7* and *CmHSP70-8* were normalized to day 1 after HTH treatment due to their undetectable basal expression at day 0, while the remaining seven genes were normalized to day 0. The four key candidate genes, *CmHSP70-4*, *CmHSP70-7*, *CmHSP70-8*, and *CmHSP70-13*, consistently showed sustained upregulation through day 3 in the tolerant cultivar JD but peaked at day 2 followed by a decline in the sensitive cultivar JTL, matching their transcriptome expression patterns. In contrast, *CmHSP70-5* and *CmHSP70-10* exhibited continuous upregulation in JTL but an early peak and decline in JD, with overall higher expression levels in JTL than in JD, suggesting that they may not be key positive regulators of HTH tolerance. The remaining genes showed distinct expression patterns: *CmHSP70-12* was sharply suppressed at day 1 in JTL with partial recovery whereas it recovered gradually in JD; *CmHSP70-14* was strongly induced at day 3 in JD but peaked earlier then declined in JTL; and CmHSP70-15 showed a fluctuating upward trend in JTL, whereas in JD it was rapidly induced at day 1 and then gradually declined. Overall, the qRT-PCR results were consistent with the transcriptome data in terms of expression trends for all nine validated genes, and the sustained high expression of the four key candidate genes in JD, in contrast to their transient induction followed by decline in JTL, is associated with the contrasting HTH responses of these cultivars and warrants functional validation before causal roles can be assigned (Figure 11).

## 4. Discussion

High temperature and humidity combined stress is a major adverse factor in melon production, which can cause protein misfolding and denaturation aggregation, seriously damaging the growth of seedlings and yield. As molecular chaperones, HSP70 proteins assist in the correct folding of proteins, scavenge excess ROS under stress, enhance the activity of the antioxidant system, and play a crucial role in plant responses to adverse stresses [33]. In this study, 16 *CmHSP70* genes were identified in melon, comparable to the family sizes of watermelon (12) [19], cucumber (12) [34], and pumpkin (21) [20]. Most CmHSP70 proteins are acidic and hydrophilic with high aliphatic indices (70.91–103.72), indicating good thermostability that is critical for maintaining protein conformation and chaperone function under HTH stress (Table 1) [35]. *CmHSP70-4/5/10* had instability indices above 40 and may be more prone to conformational changes for rapid functional deployment under stress [36]. Four *CmHSP70* members were predicted to be chloroplast-targeted by WoLF PSORT, which may be important for protecting PSII and maintaining photosynthetic homeostasis under HTH stress; two were predicted to be ER-localized and may participate in secretory protein quality control. It should be noted that these subcellular localizations are computational predictions and require experimental validation; nevertheless, this multi-compartment pattern is consistent with those reported in other plant HSP70 families (Table 1) [37].

Phylogenetic analysis classified the 16 CmHSP70 proteins into five subfamilies, consistent with the *Arabidopsis HSP70* superfamily grouping [18]. CmHSP70 proteins clustered tightly with homologs from pumpkin, cucumber, and watermelon but were more distantly related to monocot HSP70s such as rice (Figure 2), suggesting that HSP70 gene family functions in HTH stress responses may be conserved among cucurbit crops [20,38]. *CmHSP70* genes distributed randomly across eight of the 12 melon chromosomes (four clustered on chromosome 1), a pattern often associated with segmental and tandem duplication events during family expansion (Figure 1). To further trace the evolutionary origin of these genes, interspecific synteny analysis was performed, which revealed that collinear pairs with pumpkin (14), cucumber (11), and watermelon (12) far exceeded those with rice (6) and Arabidopsis (3) (Figure 5); such higher collinearity conservation within cucurbits has also been reported in pumpkin [20] and cucumber [34] *HSP70* family studies, providing a basis for mining conserved heat-responsive orthologs across cucurbit crops through comparative genomics. While the coding sequences of *CmHSP70s* are evolutionarily conserved, their exon–intron structures have diverged considerably: *CmHSP70* genes showed substantial intron number variation (0–14), with *CmHSP70-7/12/13/14* containing only one intron (Figure 3D); this compact architecture may enable rapid transcriptional activation under HTH stress [39]. Such structural diversification among family members is typically driven by gene duplication events, which provide the raw material for functional divergence. In the *CmHSP70* gene family, however, only one segmental duplication pair (*CmHSP70-2/CmHSP70-14*) was identified (Figure 6A), and all paralogous pairs had Ka/Ks < 1 (Figure 6B), indicating strong purifying selection under long-term heat and other stress pressures, with highly conserved core chaperone function [37].

Cis-acting element analysis revealed that all *CmHSP70* promoters contained light-responsive elements (up to 24 in *CmHSP70-10*) (Figure 4B), consistent with the protected-cropping environment where HTH stress is often accompanied by high light; such elements are also prevalent in cassava *MeHSP70* promoters [40]. Hormone-responsive elements including Abscisic acid, MeJA, Salicylic acid, Auxin, and Gibberellin were enriched in several *CmHSP70* promoters, most notably *CmHSP70-6*, *CmHSP70-10*, and *CmHSP70-15*, consistent with the prominent Abscisic acid and MeJA elements reported in cucumber *CsHSP70* promoters [34]. ABA and JA modulate HSF activity during heat shock, and crosstalk between hormone signaling and the HSF-HSP pathway operates under combined HTH stress [41]. Stress-related elements including Anaerobic, Low-temperature, MYB Binding Site, Wound, and Mixed stress were also detected in multiple *CmHSP70* promoters. At the transcriptional level, heat-stress induction of HSP genes is primarily governed by heat-stress transcription factors (HSFs). HSFs act as central molecular rheostats that rapidly induce HSP expression via HSE binding, while *HSP70* negatively feeds back on HSF activity, forming an intricate regulatory circuit [35]. In addition to nuclear transcriptional regulation, chloroplasts are among the most HTH-sensitive organelles, with PSII as the primary injury site [42], the four chloroplast-targeted CmHSP70 members and their light-rich promoters suggest roles in chloroplast proteostasis and photosystem protection under stress, and the overexpression of a plastid HSC70 enhances stress tolerance via ROS scavenging [43].

Predicted 3D structures and the STRING-derived protein–protein interaction network further suggest potential functional conservation and divergence of the CmHSP70 family at the structural and systems levels. As noted by Daugaard et al. in their comprehensive review of the *HSP70* family, highly homologous *HSP70* members often exhibit both functional conservation and divergence [5]. Consistent with the canonical *HSP70* structures reported in *Arabidopsis* [18] and rice [44], the majority of CmHSP70 proteins retain the dual-domain architecture, with CmHSP70-16 exhibiting a chimeric arrangement (Figure 9). The CmHSP70 interaction network exhibits a typical hierarchical modular organization, with hub genes occupying central positions and possibly mediating synergistic interactions among members, consistent with the findings in rice [45] and the general principles of HSP70 chaperone networks [46] (Figure 10). In line with this organizational pattern, the four key genes responsive to HTH stress identified in our expression analysis—*CmHSP70-4*, *7*, *8*, and *13*—are not isolated in the interaction network but are instead distributed across both functional modules: *CmHSP70-4*, *CmHSP70-7*, and *CmHSP70-13* belong to Module 1 (with *CmHSP70-4* itself being a hub gene), while *CmHSP70-8* belongs to Module 2. This distribution pattern suggests that the HTH stress response may not be mediated by an isolated stress-specific subnetwork but rather may rely on the coordinated activation of key members embedded within the core chaperone interaction architecture, consistent with the report in Arabidopsis that inducible *HSP70s* function in concert with constitutive members to amplify chaperone capacity [18] and Kozeko’s proposition that inducible and constitutive *HSP70s* play complementary roles in stress responses [47]. Notably, *CmHSP70-16* harbors a chimeric domain architecture: its canonical C-terminal substrate-binding domain is replaced by a DUF4283 domain and the N-terminal ATPase domain is truncated. Since the C-terminal substrate-binding domain and its EEVD motif mediate substrate recognition and co-chaperone binding [48], this domain replacement likely abolishes its interactions with other *HSP70* network members, likely accounting for its isolated node in the PPI network. The DUF4283 domain and the associated extended flexible C-terminal region may instead confer a unique client protein repertoire or subcellular localization, reflecting functional divergence from the canonical *HSP70* chaperone network [49]. In this study, the convergence of structural conservation, interaction network topology, and expression profiles strengthens the candidacy of *CmHSP70-4/7/8/13* as priority targets for functional validation. Among them, *CmHSP70-4* also occupies a hub position in the interaction network, while the other hub genes *CmHSP70-5/10/15* may serve as important modulators that fine-tune the chaperone response under HTH stress.

Tissue-expression profiling based on public transcriptome data showed that *CmHSP70-4/11/15* were highly expressed across root, stem, leaf, flower, and fruit, *CmHSP70-13* was broadly expressed with the highest levels in fruit, male flowers, and leaves, while *CmHSP70-1/6/8/9/12/14/16* showed low or no expression in all tissues (Figure 7). Notably, *CmHSP70-2*, which showed tissue-specific low-to-moderate expression in public tissue-expression data, exhibited basal expression in the 0-day controls of both cultivars and was significantly upregulated under HTH stress, indicating that this gene is not transcriptionally silent but a stress-inducible gene with basal expression [47]. *CmHSP70-13*, by contrast, was already expressed in multiple tissues in public data (particularly fruit and male flowers) and was further strongly induced under HTH stress, suggesting that it functions in both reproductive development and stress responses. HTH stress transcriptome profiling classified the 16 *CmHSP70* members into four expression patterns: extremely low expression, constitutive expression, high basal expression, and strong induction, with *CmHSP70-4/7/8/13* as the core HTH-responsive genes (Figure 8); such partitioning between constitutive and inducible members has been reported in Arabidopsis [18]. The two cultivars showed distinct temporal dynamics: *CmHSP70-4*, *CmHSP70-7*, *CmHSP70-8*, and *CmHSP70-13* showed sustained upregulation through day 3 in JD but peaked at day 2 then declined in JTL; similar divergence has been observed between heat-tolerant and sensitive sorghum genotypes [50], and the sustained rather than transient induction of these four genes in JD is consistent with the notion that durable HSP70 responsiveness may contribute to HTH tolerance, although this remains to be confirmed in a broader panel of genotypes [47]. qRT-PCR validated the transcriptome findings, confirming sustained upregulation of *CmHSP70-4*, *CmHSP70-7*, *CmHSP70-8*, and *CmHSP70-13* in JD and contrasting temporal dynamics across cultivars (Figure 11), a pattern also reported in rice heat responses [44]. These findings nominate CmHSP70-4/7/8/13 as candidate genes for future functional studies and may inform molecular breeding efforts for HTH tolerance in melon, although validation in additional genetic backgrounds is needed. In addition, in species such as *Solanum tuberosum* [17], barley [51], multiple *HSP70* genes are significantly upregulated or downregulated under high temperature, indicating that *HSP70* has relatively conserved functions in abiotic stresses such as high temperature. Systematic studies on the functions of *CmHSP70* will help reveal the molecular basis of CmHSP70-mediated abiotic stress responses in melon.

## 5. Conclusions

During summer cultivation, melon frequently suffers from combined high temperature and humidity (HTH) stress, which causes protein misfolding, photosystem damage, and excessive ROS accumulation. As central molecular chaperones, HSP70s play a pivotal role in refolding denatured proteins and maintaining cellular proteostasis under such stress conditions. In this study, we present the first genome-wide characterization of the HSP70 gene family in melon and link its members to combined high temperature and humidity (HTH) stress responses. The 16 identified *CmHSP70* genes, phylogenetically grouped into five subfamilies with the closest affinity to cucurbit crops, exhibit evolutionary conservation in coding sequences under purifying selection yet functional diversification in gene structures, cis-regulatory elements, and computationally predicted subcellular localizations. Notably, the broad enrichment of stress- and hormone-responsive cis-elements in their promoters, together with their computationally predicted multi-compartment subcellular distributions, generates hypotheses regarding how these genes may perceive and respond to combined HTH-stress signals across different cellular sites. The most salient finding is that four *CmHSP70* genes (*CmHSP70-4*, *CmHSP70-7*, *CmHSP70-8*, and *CmHSP70-13*) exhibit genotype-specific temporal expression dynamics, with sustained upregulation through day 3 in the HTH-tolerant cultivar JD and transient induction followed by decline in the sensitive JTL. This contrast suggests that durable HSP70 induction, rather than mere stress responsiveness, is associated with differential HTH-stress performance between the two tested melon genotypes. The integration of transcriptomic profiling, qRT-PCR validation, three-dimensional structural modeling, and protein interaction network analysis collectively strengthens the candidacy of these four genes as priority targets for functional validation. Future work should focus on overexpression or knockout studies to directly establish the causal roles of CmHSP70-4/7/8/13 in HTH tolerance, and on exploring their utility as molecular markers for breeding stress-tolerant melon and other cucurbits.

## Figures and Tables

**Table 1:** Physicochemical properties and subcellular localization of *CmHSP70* genes in melon.

Gene Name	Gene ID	AA	MW (kDa)	pI	II	AI	GRAVY	Localization
*CmHSP70-1*	*MELO3C028818*	121	13.50	7.77	34.06	70.91	−0.386	Extracellular
*CmHSP70-2*	*MELO3C024086*	677	72.95	5.03	29.21	88.55	−0.281	Chloroplast
*CmHSP70-3*	*MELO3C021100*	201	22.12	5.74	26.82	86.37	−0.22	Chloroplast
*CmHSP70-4*	*MELO3C024230*	844	92.87	5.37	41.27	80.56	−0.421	Cytoplasm
*CmHSP70-5*	*MELO3C026698*	865	97.20	5.63	46.35	85.33	−0.324	Nucleus
*CmHSP70-6*	*MELO3C020341*	705	75.31	5.19	33.29	87.13	−0.289	Chloroplast
*CmHSP70-7*	*MELO3C003917*	650	71.28	5.15	35.07	84.17	−0.41	Cytoplasm
*CmHSP70-8*	*MELO3C006536*	604	65.73	5.64	38.28	96.08	0.061	Chloroplast
*CmHSP70-9*	*MELO3C031721*	94	10.26	6.18	26.46	103.72	0.187	Cytoplasm
*CmHSP70-10*	*MELO3C026399*	898	99.93	5.42	43.53	82.42	−0.498	ER
*CmHSP70-11*	*MELO3C018045*	607	65.31	5.34	37.21	88.4	−0.326	Cytoplasm
*CmHSP70-12*	*MELO3C003119*	647	70.88	5.16	34.67	83.52	−0.407	Cytoplasm
*CmHSP70-13*	*MELO3C021648*	648	71.08	5.21	34.58	82.95	−0.411	Cytoplasm
*CmHSP70-14*	*MELO3C021658*	652	71.51	5.10	36.29	81.52	−0.442	Cytoplasm
*CmHSP70-15*	*MELO3C005757*	665	73.47	5.10	28.33	86.96	−0.461	ER
*CmHSP70-16*	*MELO3C035452*	326	35.78	9.71	33.25	94.2	−0.276	Cytoplasm

Note: AA, number of amino acids; MW, molecular weight; pI, isoelectric point; II, instability index; AI, aliphatic index; GRAVY, grand average of hydropathicity; ER, Endoplasmic reticulum.

## Data Availability

The RNA-seq data used in this study are available in the NCBI Sequence Read Archive (SRA) under accession number PRJNA1434721 (https://www.ncbi.nlm.nih.gov/bioproject/PRJNA1434721). All other data generated or analyzed during this study are included in this published article and its Supplementary Materials.

## Supplementary Materials

The following supporting information can be downloaded at https://www.mdpi.com/article/10.3390/genes17101257/s1. File S1: Protein sequences of 16 CmHSP70 family members in melon; Table S1: Pair-specific Ka, Ks, and Ka/Ks values for paralogous CmHSP70 pairs analyzed in melon; Table S2: Detailed STRING mapping and network attributes of CmHSP70 proteins; Table S3: Primer sequences for qRT-PCR analysis; Table S4: Two-way ANOVA of log_2_(FPKM + 1)-transformed expression values of the *CmHSP70* genes under HTH stress; Table S5: Conserved domain architecture, coordinates, coverage, and classification of the 16 CmHSP70 proteins in melon.
