# Supplementary material for: Identification and Expression Profiling of the HSP70 Gene Family in Melon Under Combined High Temperature and Humidity Stress
Source: Genes (Basel). 2026 Oct 11;17(10):1257. doi: 10.3390/genes17101257 (PMC13704437; doi:10.3390/genes17101257)
Supplement: Supplementary file 1 [file genes-17-01257-s001.zip › Supplementary Table S1. Pair-specific Ka, Ks, and KaKs values for paralogous CmHSP70 pairs analyzed in melon..pdf]

Supplementary Table S1. Pair-specific Ka, Ks, and Ka/Ks values for paralogous CmHSP70 pairs analyzed in melon.

A total of 62 paralogous gene pairs with reliable Ka and Ks estimates were analyzed. All Ka/Ks ratios were < 1, indicating purifying selection. These 62 data points correspond to Figure 6B. The single MCScanX segmental duplication pair CmHSP70-2/CmHSP70-14 had Ka = 0.4584, Ks = 2.2727, Ka/Ks = 0.2017.

Gene pair	Ka	Ks	Ka/Ks	Selection
CmHSP70-1/CmHSP70-2	0.5831	2.3706	0.2460	Purifying (Ka/Ks < 1)
CmHSP70-1/CmHSP70-3	0.2517	0.3896	0.6462	Purifying (Ka/Ks < 1)
CmHSP70-1/CmHSP70-5	0.8447	1.9524	0.4326	Purifying (Ka/Ks < 1)
CmHSP70-1/CmHSP70-6	0.6109	2.8333	0.2156	Purifying (Ka/Ks < 1)
CmHSP70-1/CmHSP70-8	0.7306	2.3805	0.3069	Purifying (Ka/Ks < 1)
CmHSP70-1/CmHSP70-10	0.9037	1.9865	0.4549	Purifying (Ka/Ks < 1)
CmHSP70-1/CmHSP70-12	0.2521	1.3018	0.1937	Purifying (Ka/Ks < 1)
CmHSP70-1/CmHSP70-14	0.2417	0.4773	0.5063	Purifying (Ka/Ks < 1)
CmHSP70-1/CmHSP70-16	0.3285	0.5264	0.6240	Purifying (Ka/Ks < 1)
CmHSP70-2/CmHSP70-9	0.3305	3.0483	0.1084	Purifying (Ka/Ks < 1)
CmHSP70-2/CmHSP70-14	0.4584	2.2727	0.2017	Purifying (Ka/Ks < 1)
CmHSP70-2/CmHSP70-15	0.4892	4.3701	0.1119	Purifying (Ka/Ks < 1)
CmHSP70-2/CmHSP70-16	1.0388	2.0823	0.4989	Purifying (Ka/Ks < 1)
CmHSP70-3/CmHSP70-4	0.6701	3.2832	0.2041	Purifying (Ka/Ks < 1)
CmHSP70-3/CmHSP70-5	0.6916	3.0614	0.2259	Purifying (Ka/Ks < 1)
CmHSP70-3/CmHSP70-6	0.4766	2.2087	0.2158	Purifying (Ka/Ks < 1)
CmHSP70-3/CmHSP70-8	0.8071	2.6351	0.3063	Purifying (Ka/Ks < 1)
CmHSP70-3/CmHSP70-10	0.8087	2.3404	0.3456	Purifying (Ka/Ks < 1)
CmHSP70-3/CmHSP70-12	0.0892	1.4390	0.0620	Purifying (Ka/Ks < 1)
CmHSP70-3/CmHSP70-14	0.0467	0.0521	0.8969	Purifying (Ka/Ks < 1)
CmHSP70-3/CmHSP70-16	0.3170	0.5193	0.6104	Purifying (Ka/Ks < 1)
CmHSP70-4/CmHSP70-6	0.8077	3.5112	0.2300	Purifying (Ka/Ks < 1)
CmHSP70-4/CmHSP70-7	0.7039	2.5214	0.2792	Purifying (Ka/Ks < 1)
CmHSP70-4/CmHSP70-8	0.9398	2.1161	0.4441	Purifying (Ka/Ks < 1)
CmHSP70-4/CmHSP70-9	0.6807	1.9896	0.3421	Purifying (Ka/Ks < 1)
CmHSP70-4/CmHSP70-12	0.6940	4.3381	0.1600	Purifying (Ka/Ks < 1)
CmHSP70-4/CmHSP70-14	0.7046	3.0154	0.2337	Purifying (Ka/Ks < 1)
CmHSP70-5/CmHSP70-6	0.8397	2.9075	0.2888	Purifying (Ka/Ks < 1)
CmHSP70-5/CmHSP70-8	0.9061	1.6989	0.5333	Purifying (Ka/Ks < 1)
CmHSP70-5/CmHSP70-9	0.6934	2.1300	0.3255	Purifying (Ka/Ks < 1)
CmHSP70-5/CmHSP70-15	0.7448	2.9558	0.2520	Purifying (Ka/Ks < 1)
CmHSP70-5/CmHSP70-16	1.0228	2.0435	0.5005	Purifying (Ka/Ks < 1)
CmHSP70-6/CmHSP70-8	0.7841	2.4634	0.3183	Purifying (Ka/Ks < 1)

CmHSP70-6/CmHSP70-9	0.3536	2.3568	0.1500	Purifying (Ka/Ks < 1)
CmHSP70-6/CmHSP70-10	0.7622	2.5964	0.2936	Purifying (Ka/Ks < 1)
CmHSP70-6/CmHSP70-11	0.4063	2.6011	0.1562	Purifying (Ka/Ks < 1)
CmHSP70-6/CmHSP70-12	0.4995	2.6062	0.1916	Purifying (Ka/Ks < 1)
CmHSP70-6/CmHSP70-14	0.4900	2.4518	0.1999	Purifying (Ka/Ks < 1)
CmHSP70-6/CmHSP70-16	0.9169	3.4478	0.2659	Purifying (Ka/Ks < 1)
CmHSP70-7/CmHSP70-8	0.6728	4.7847	0.1406	Purifying (Ka/Ks < 1)
CmHSP70-7/CmHSP70-16	0.4710	1.7457	0.2698	Purifying (Ka/Ks < 1)
CmHSP70-8/CmHSP70-9	0.8411	1.5715	0.5352	Purifying (Ka/Ks < 1)
CmHSP70-8/CmHSP70-10	0.9135	2.0193	0.4524	Purifying (Ka/Ks < 1)
CmHSP70-8/CmHSP70-12	0.6748	2.7939	0.2415	Purifying (Ka/Ks < 1)
CmHSP70-8/CmHSP70-13	0.6482	3.5255	0.1839	Purifying (Ka/Ks < 1)
CmHSP70-8/CmHSP70-14	0.6798	2.6346	0.2580	Purifying (Ka/Ks < 1)
CmHSP70-8/CmHSP70-15	0.7248	3.2450	0.2234	Purifying (Ka/Ks < 1)
CmHSP70-9/CmHSP70-10	0.7605	2.1312	0.3569	Purifying (Ka/Ks < 1)
CmHSP70-9/CmHSP70-11	0.3289	2.7991	0.1175	Purifying (Ka/Ks < 1)
CmHSP70-9/CmHSP70-12	0.1121	1.0054	0.1115	Purifying (Ka/Ks < 1)
CmHSP70-10/CmHSP70-11	0.7609	2.0193	0.3768	Purifying (Ka/Ks < 1)
CmHSP70-10/CmHSP70-14	0.7146	2.9757	0.2402	Purifying (Ka/Ks < 1)
CmHSP70-10/CmHSP70-16	0.8737	1.9282	0.4531	Purifying (Ka/Ks < 1)
CmHSP70-11/CmHSP70-15	0.4436	2.1310	0.2081	Purifying (Ka/Ks < 1)
CmHSP70-11/CmHSP70-16	0.8814	3.2025	0.2752	Purifying (Ka/Ks < 1)
CmHSP70-12/CmHSP70-13	0.0322	2.4642	0.0131	Purifying (Ka/Ks < 1)
CmHSP70-12/CmHSP70-14	0.0368	1.8252	0.0202	Purifying (Ka/Ks < 1)
CmHSP70-12/CmHSP70-15	0.3044	1.6216	0.1877	Purifying (Ka/Ks < 1)
CmHSP70-12/CmHSP70-16	0.3033	1.2401	0.2446	Purifying (Ka/Ks < 1)
CmHSP70-13/CmHSP70-14	0.0397	2.3451	0.0169	Purifying (Ka/Ks < 1)
CmHSP70-13/CmHSP70-16	0.3000	2.2399	0.1339	Purifying (Ka/Ks < 1)
CmHSP70-14/CmHSP70-16	0.2782	0.6228	0.4468	Purifying (Ka/Ks < 1)
