# Supplementary material for: Identification and Expression Profiling of the HSP70 Gene Family in Melon Under Combined High Temperature and Humidity Stress
Source: Genes (Basel). 2026 Oct 11;17(10):1257. doi: 10.3390/genes17101257 (PMC13704437; doi:10.3390/genes17101257)
Supplement: Supplementary file 1 [file genes-17-01257-s001.zip › Supplementary Table S2. Detailed STRING mapping and network attributes of CmHSP70 proteins..pdf]

Supplementary Table S2. Detailed STRING mapping and network attributes of CmHSP70 proteins.

The protein-protein interaction network was constructed using the STRING database (v12.0) against the Cucumis melo proteome, with a medium confidence score threshold of 0.4. Interactions were derived from combined evidence channels including curated databases, experimental determinations, and text mining. Hub genes were defined as nodes with degree values ≥ 10, which exceeds the network average degree of 5.86.

No.	CmHSP70 Gene	STRING ID	Identity (%)	Mapping Type	Module	Hub
**1**	CmHSP70-1	1194695.A0A5A7TM26	93.2	Same node (with CmHSP70-14)	Module 1	No
**2**	CmHSP70-2	1194695.A0A5A7TDI2	88.0	Same node (with CmHSP70-6)	Module 2	No
**3**	CmHSP70-3	1194695.A0A5A7SN84	92.5	Unique	Module 1	No
**4**	CmHSP70-4	1194695.A0A5D3BXZ3	98.3	Unique	Module 1	Yes (Degree=10)
**5**	CmHSP70-5	1194695.A0A5D3DVS3	97.2	Unique	Module 1	Yes (Degree=10)
**6**	CmHSP70-6	1194695.A0A5A7TDI2	100.0	Same node (with CmHSP70-2)	Module 2	No
**7**	CmHSP70-7	1194695.A0A5A7TXD4	100.0	Unique	Module 1	No
**8**	CmHSP70-8	1194695.A0A5D3D052	99.8	Unique	Module 2	No
**9**	CmHSP70-9	1194695.A0A5A7TWV4	100.0	Unique	Module 1	No
**10**	CmHSP70-10	1194695.A0A5D3DXU5	100.0	Unique	Module 2	Yes (Degree=10)
**11**	CmHSP70-11	1194695.A0A5A7TLA5	100.0	Unique	Module 2	No
**12**	CmHSP70-12	1194695.A0A5A7SRF3	100.0	Unique	Module 1	No
**13**	CmHSP70-13	1194695.A0A5D3DHM6	100.0	Unique	Module 1	No
**14**	CmHSP70-14	1194695.A0A5A7TM26	99.8	Same node (with CmHSP70-1)	Module 1	No
**15**	CmHSP70-15	1194695.A0A5A7TL36	100.0	Unique	Module 1	Yes (Degree=12)
**16**	CmHSP70-16	1194695.A0A5A7TK34	70.3	Isolated	Isolated	No
