# Supplementary material for: Identification and Expression Profiling of the HSP70 Gene Family in Melon Under Combined High Temperature and Humidity Stress
Source: Genes (Basel). 2026 Oct 11;17(10):1257. doi: 10.3390/genes17101257 (PMC13704437; doi:10.3390/genes17101257)
Supplement: Supplementary file 1 [file genes-17-01257-s001.zip › Supplementary Table S3. Primer sequences for qRT-PCR analysis.pdf]

Supplementary Table S3. Primer sequences for qRT-PCR analysis

Gene name	Forward Primer (5'-3')	Reverse Primer (5'-3')	R^2	Efficiency (%)
CmHSP70-4	GACCCGATAGAGGAGCGTTACA	GGTAGTGAGTCTTGGTGCTGCT	0.999	100.1
CmHSP70-5	TCCTGCCGCCGTTACCTTCT	CACTCATCTCTGCTTGCCTGGT	0.9999	101.8
CmHSP70-7	GGACCAGGAGACAAGCCGATGA	GCCGCCGTTGGTTCATTGATG	0.9999	101.3
CmHSP70-8	AGTAGTGGTTGGCGGATGTTCA	AGGCGTTGCTTGGATGCTAAGA	0.998	94.5
CmHSP70-10	GGAAGACTGTGTCGGTCAACCA	TTTGCCATCGCCTTGGGATAGT	0.999	102.2
CmHSP70-12	GCCGTTGTCACTGTTCCTGCTT	TCACCGACACTTGTTGCCTTCT	0.999	106.3
CmHSP70-13	TGACGCTGCCAAGAACCAAGT	TCACGGAGGAACCAAGGTAAGC	0.999	98.8
CmHSP70-14	CTGAGGCGTATCTCGGCACAAC	GCCAGAGCTGCTCGACTTCTTG	0.9999	98.0
CmHSP70-15	TTGAGTGGAGAAGGCGGTGAAG	ACGGTGGTCTGTTGATCTTGGT	0.999	95.3
Actin	TCTATTCCAGCCATCTCTC	GACCCTCCAATCCAAAC	0.999	98.8
