# Supplementary material for: Identification and Expression Profiling of the HSP70 Gene Family in Melon Under Combined High Temperature and Humidity Stress
Source: Genes (Basel). 2026 Oct 11;17(10):1257. doi: 10.3390/genes17101257 (PMC13704437; doi:10.3390/genes17101257)
Supplement: Supplementary file 1 [file genes-17-01257-s001.zip › Supplementary Table S4. Two-way ANOVA of log2(FPKM+1)-transformed expression values of the CmHSP70 genes under HTH stress..pdf]

Supplementary Table S4. Two-way ANOVA of log_2(FPKM+1)-transformed expression values of the CmHSP70 genes under HTH stress

Gene	Gene ID	Cultivar F	Cultivar P	Time F	Time P	Interaction F	Interaction P	Note
CmHSP70-1	MELO3C028818	NA	NA	NA	NA	NA	NA	Excluded (max FPKM < 1)
CmHSP70-2	MELO3C024086	7.31	0.0156	701.04	<0.0001	3.57	0.0376	Significant interaction
CmHSP70-3	MELO3C021100	38.33	<0.0001	5.21	0.0106	16.72	<0.0001	Significant interaction
CmHSP70-4	MELO3C024230	6.45	0.0219	13.85	0.0001	6.69	0.0039	Significant interaction
CmHSP70-5	MELO3C026698	0.02	0.9017	17.71	<0.0001	6.50	0.0044	Significant interaction
CmHSP70-6	MELO3C020341	7.77	0.0132	0.51	0.6780	2.00	0.1552	Non-significant interaction
CmHSP70-7	MELO3C003917	4.79	0.0438	157.72	<0.0001	2.57	0.0907	Marginal interaction (P = 0.091)
CmHSP70-8	MELO3C006536	15.56	0.0012	161.18	<0.0001	6.61	0.0041	Significant interaction
CmHSP70-9	MELO3C031721	NA	NA	NA	NA	NA	NA	Excluded (max FPKM < 1)
CmHSP70-10	MELO3C026399	0.01	0.9389	7.34	0.0026	4.42	0.0192	Significant interaction
CmHSP70-11	MELO3C018045	1.88	0.1889	9.73	0.0007	5.01	0.0122	Significant interaction
CmHSP70-12	MELO3C003119	10.02	0.0060	10.96	0.0004	9.24	0.0009	Significant interaction
CmHSP70-13	MELO3C021648	29.99	0.0001	133.32	<0.0001	6.99	0.0032	Significant interaction
CmHSP70-14	MELO3C021658	29.19	0.0001	13.23	0.0001	16.55	<0.0001	Significant interaction
CmHSP70-15	MELO3C005757	0.08	0.7872	16.25	<0.0001	2.24	0.1228	Non-significant interaction
CmHSP70-16	MELO3C035452	NA	NA	NA	NA	NA	NA	Excluded (max FPKM < 1)

Note: Cultivar = effect of cultivar (JD vs. JTL); Time = effect of treatment time (0-3 days); Interaction = cultivar x time interaction. Among the 13 assessable genes, 10 showed a significant cultivar x time interaction (P < 0.05; CmHSP70-2, -3, -4, -5, -8, -10, -11, -12, -13, -14), one was marginal (CmHSP70-7, P = 0.091), and two were non-significant (CmHSP70-6 and -15). CmHSP70-1, -9, and -16 were excluded because their maximum FPKM was below 1.
