# Supplementary material for: Identification and Expression Profiling of the HSP70 Gene Family in Melon Under Combined High Temperature and Humidity Stress
Source: Genes (Basel). 2026 Oct 11;17(10):1257. doi: 10.3390/genes17101257 (PMC13704437; doi:10.3390/genes17101257)
Supplement: Supplementary file 1 [file genes-17-01257-s001.zip › Supplementary Table S5. Conserved domain architecture, coordinates, coverage, and classification of the 16 CmHSP70 proteins in melon.pdf]

Supplementary Table S5. Conserved domain architecture, coordinates, coverage, and classification of the 16 CmHSP70 proteins in melon

Conserved domain verification of the 16 CmHSP70 proteins, including domain architecture, coordinates, coverage, and classification; identification thresholds are stated below.

Gene name	Domain architecture	Domain coordinates (aa)	Coverage (%)	Classification
CmHSP70-1	Partial HSP70-related domains	12-83	59.5	Truncated/partial HSP70-like
CmHSP70-2	Canonical HSP70 domains	45-639	87.9	Canonical HSP70
CmHSP70-3	Partial HSP70-related domains	9-192	91.5	Truncated/partial HSP70-like
CmHSP70-4	Canonical HSP70 domains	3-691	81.6	Canonical HSP70
CmHSP70-5	Canonical HSP70 domains	87-755	77.3	Canonical HSP70
CmHSP70-6	Canonical HSP70 domains	73-667	84.4	Canonical HSP70
CmHSP70-7	Canonical HSP70 domains	9-618	93.8	Canonical HSP70
CmHSP70-8	Canonical HSP70 domains	61-554	81.8	Canonical HSP70
CmHSP70-9	Partial HSP70-related domains	1-85	90.4	Truncated/partial HSP70-like
CmHSP70-10	Canonical HSP70 domains	29-741	79.4	Canonical HSP70
CmHSP70-11	Canonical HSP70 domains	1-578	95.2	Canonical HSP70
CmHSP70-12	Canonical HSP70 domains	9-618	94.3	Canonical HSP70
CmHSP70-13	Canonical HSP70 domains	9-618	94.1	Canonical HSP70
CmHSP70-14	Canonical HSP70 domains	9-618	93.6	Canonical HSP70
CmHSP70-15	Canonical HSP70 domains	38-645	91.4	Canonical HSP70
CmHSP70-16	ASKHA_ATPase-like + DUF4283	203-283 (DUF4283)<br>283-315 (ASKHA)	35.0	HSP70-related chimeric

Note: Domain architecture is based on NCBI Batch Conserved Domain Database (CDD, Batch CD-search) hits. "Canonical HSP70 domains" indicates a continuous match covering the complete HSP70 domain (approximately 490-710 aa; CDD Incomplete = -). "Partial HSP70-related domains" indicates that only a short fragment of the ASKHA_ATPase-like superfamily is present (CmHSP70-1, -3, and -9: 72, 184, and 85 aa; Incomplete = C). CmHSP70-16 carries an ASKHA_ATPase-like fragment fused to a DUF4283 domain. Coverage = total length of the matched region(s) / protein length × 100%.

Identification thresholds: HMMER, E-value cutoff = 1e-5; BLASTP, E-value < 1e-5. All candidate sequences were then confirmed with the NCBI Conserved Domain Database (CDD) and SMART.
